# AMXT-1501 targets membrane phospholipids against Gram-positive and -negative multidrug-resistant bacteria

**DOI:** 10.1080/22221751.2024.2321981

**Published:** 2024-02-29

**Authors:** Jinxin Zheng, Xiaoju Liu, Yanpeng Xiong, Qingyin Meng, Peiyu Li, Fan Zhang, Xiaoming Liu, Zhiwei Lin, Qiwen Deng, Zewen Wen, Zhijian Yu

**Affiliations:** a Department of Infectious Diseases and Shenzhen Key Lab of Endogenous Infection, Shenzhen Nanshan People’s Hospital and the 6th Affiliated Hospital of Shenzhen University Medical School, Shenzhen, People’s Republic of China; b Department of Tuberculosis, Shenzhen Nanshan Center for Chronic Disease Control, Shenzhen, People’s Republic of China; c Department of Gastroenterology, Shenzhen Qianhai Shekou Free Trade Zone Hospital, Shenzhen, People’s Republic of China

**Keywords:** Multidrug-resistant, MRSA, ESBL, CRE, AMXT-1501, Cardiolipin, Phosphatidylglycerol

## Abstract

The rapid proliferation of multidrug-resistant (MDR) bacterial pathogens poses a serious threat to healthcare worldwide. Carbapenem-resistant (CR) Enterobacteriaceae, which have near-universal resistance to available antimicrobials, represent a particularly concerning issue. Herein, we report the identification of AMXT-1501, a polyamine transport system inhibitor with antibacterial activity against Gram-positive and -negative MDR bacteria. We observed minimum inhibitory concentration (MIC)_50_/MIC_90_ values for AMXT-1501 in the range of 3.13–12.5 μM (2.24–8.93 μg /mL), including for methicillin-resistant *Staphylococcus aureus* (MRSA), CR *Escherichia coli*, *Klebsiella pneumoniae*, and *Pseudomonas aeruginosa*. AMXT-1501 was more effective against MRSA and CR *E. coli* than vancomycin and tigecycline, respectively. Subinhibitory concentrations of AMXT-1501 reduced the biofilm formation of *S. aureus* and *Enterococcus faecalis*. Mechanistically, AMXT-1501 exposure damaged microbial membranes and increased membrane permeability and membrane potential by binding to cardiolipin (CL) and phosphatidylglycerol (PG). Importantly, AMXT-1501 pressure did not induce resistance readily in the tested pathogens.

## Introduction

Since the introduction of penicillin in 1928, diverse types of antibiotics have been developed for bacterial infection treatment. However, widespread use and frequent overuse of antibiotics have been selected for progressively stronger bacterial resistance rates [1]. *Staphylococcus aureus* is a pathogenic species of Gram-positive bacteria. Since the first strain of methicillin-resistant *S. aureus* (MRSA) was isolated in 1961, rates of MRSA infection have increased rapidly and MRSA strains have become difficult-to-eradicate nosocomial pathogens due to their resistance to multiple antibiotics [2]. There are increasing reports of clinically isolated MRSA strains that are insensitive or resistant to the antimicrobials most commonly used to treat MRSA infections, namely vancomycin, linezolid, and daptomycin [3–5].

Biofilm formation by Gram-positive bacteria, including staphylococcus species and enterococcus species, is another challenging issue that decreases the effectiveness of anti-infection treatments [6–8]. Bacterial biofilm is an extracellular membranous material composed of polysaccharide adhesion molecules, proteins, phospholipids, and extracellular DNA that protects bacteria by reducing their sensitivity to antimicrobials and helping them to evade host immune cell attacks and phagocytosis, thereby extending infection and delaying healing [9, 10]. Although biofilm formation augments infection persistence, most first-line antimicrobials do not inhibit biofilm formation effectively and nor do they eradicate established biofilms [6–10].

The most commonly clinically isolated pathogenic bacteria are Gram-negative Enterobacteriaceae, such as *Escherichia coli* and *Klebsiella pneumoniae* [11, 12]. The widespread use and abuse of antimicrobials have led to ever-increasing occurrences of drug-resistant Enterobacteriaceae, including extended-spectrum beta-lactamase (ESBL)-producing Enterobacteriaceae [13]. Carbapenem antimicrobials, such as meropenem, are currently the first-line clinical treatment for ESBL-producing Enterobacteriaceae infections but the emergence of carbapenem-resistant (CR) Enterobacteriaceae is making ESBL-producing Enterobacteriaceae infections more difficult to treat [14–16].

Antimicrobial resistance is underlying a new public health crisis [17]. In 2019, there were 1.27 million deaths related to multidrug resistant (MDR) bacterial infections worldwide, and the World Health Organization predicts that there will be 10 million such deaths per year by 2050 [18]. There is an urgent need for the development of new broad-spectrum antimicrobials, especially for agents that are effective against CR and MDR Enterobacteriaceae infections. Thus this study aims to screen chemicals with broad-spectrum antibacterial activity, especially those with strong antibacterial activity against MDR bacteria, from the MedChemExpress Library, and explore their potential targets in MDR bacteria. Interestingly, this study explored that AMXT-1501 from MedChemExpress Library, a novel polyamine transport inhibitor, synergizes with alpha-difluoromethylornithine (DFMO) in inhibiting neuroblastoma cell proliferation [19], has powerful antibacterial activity against Gram-positive and -negative MDR bacteria. Therefore, this study focuses on AMXT-1501 and explores its possible antibacterial mechanism.

## Materials and methods

### Bacterial strains and growth conditions

The reference strains *S. aureus* ATCC29213, *S. aureus* SA113 (ATCC35556), and *E. coli* ATCC25922 were purchased from American Type Culture Collection. The reference strain *K. pneumoniae* NTUH-K2044 was obtained from Jin-Town Wang and Zhi-Rong Lin (Department of Microbiology, National Taiwan University College of Medicine, Taipei, Taiwan, and Department of Internal Medicine, National Taiwan University Hospital, Taipei, Taiwan). A collection of 126 *S. aureus*, 32 *S. epidermids*, 49 *E. faecium*, 71 *E. faecalis*, 184 E. coli, 162 *K. pneumoniae*, 36 *A. baumanni*, and 40 *P. aeruginosa* clinical isolates were obtained from the Shenzhen Nanshan People’s Hospital and the 6th Affiliated Hospital of Shenzhen University Medical School (Grade A, level III Hospital, 1500 beds) between January 1 2015 and December 31 2021. The clinical isolates were identified with a Phoenix 100 automated microbiology system (BD, Franklin Lakes, NJ, USA) and then two subcultured generations were re-identified with matrix-assisted laser desorption ionization time-of-flight mass spectrometry (IVD MALDI Biotyper, Germany).

*S. aureus*, *S. epidermids*, *E. faecium*, and *E. faecalis* were grown in TSB (tryptic soy broth) at 37 °C with shaking unless otherwise stated. *E. coli*, *K. pneumoniae*, *A. baumannii*, and *P. aeruginosa* were grown in Luria–Bertani medium at 37 °C with shaking unless otherwise stated. For antimicrobial susceptibility testing and time-kill assay, strains were grown in cation-adjusted Mueller-Hinton broth (CAMHB) at 37 °C with shaking. For biofilm assays, *S. aureus* and *E. faecalis* were grown in TSB with 0.5% glucose (TSBG) at 37 °C under static incubation.

### Antimicrobials and chemicals

Oxacillin sodium (HY-B0465; 99.55%), ampicillin sodium (HY-B0522A; ≥ 98%), cefazolin sodium (HY-B1078; 95.45%), ceftriaxone sodium (HY-B0712B; 98.03%), cefepime (HY-B0692; 99.78%), imipenem (HY-B1369A; ≥ 98%), meropenem (HY-13678; ≥ 98%), levofloxacin hydrochloride (HY-B0330B; ≥ 98%), erythromycin (HY-B0220; 99.86%), azithromycin (HY-17506; ≥ 98%), doxycycline (HY-N0565; ≥ 98%), tigecycline hydrochloride (HY-B0117A; ≥ 98%), gentamicin (HY-A0276A; ≥ 98%), linezolid (HY-10394; 99.95%), daptomycin (HY-B0108; 99.90%), colistin (HY-113678; ≥ 98%), vancomycin (HY-B0671; 96.66%) were purchased from MedChemExpress (Shanghai, China). AMXT-1501 tetrahydrochloride (HY-124617A; ≥ 98%) was purchased from MedChemExpress (Shanghai, China) and was dissolved in sterile dddH_2_O in this study.

Chemicals Screening Library [HY-LD-000002869; 2291 chemicals; ≥ 98%; Classification of Chemicals (based on pathway): Cell Cycle/DNA Damage, 252; Apoptosis, 255; Autophagy, 63; Cytoskeleton, 12; Epigenetics, 170; GPCR/G Protein, 230; Immunology/Inflammation, 85; JAK/STAT Signaling, 48; MAPK/ERK Pathway, 53; Membrane Transporter/Ion Channel, 110; Metabolic Enzyme/Protease, 292; Neuronal Signaling, 38; NF-κB, 15; PI3 K/Akt/mTOR, 58; PROTAC, 33; Protein Tyrosine Kinase/RTK, 111; Stem Cell/Wnt, 28; TGF-beta/Smad, 13; Others, 425] was purchased from MedChemExpress (Shanghai, China).

Spermine (S425636; 10 mM in water), spermidine [S421040; 10 mM in dimethyl sulfoxide (DMSO)], 1,4-diaminobutane dihydrochloride (D423441; 10 mM in water), and 1,5-diaminopentane dihydrochloride (D421724; 10 mM in DMSO) were purchased from Aladdin (Aladdin, Shanghai, China). CL (841199P; ≥ 99%), phosphatidylcholine (840051P; ≥ 99%) (L130332), phosphatidylethanolamine (840027P; ≥ 99%), PG (841188P; ≥ 99%), LPS (*E. coli*; L2630), peptidoglycan (*S. aureus;* 77140), propidium iodide (P4170; ≥ 94%), and DiBAC4(3) (44977; ≥ 98%) were purchased from Sigma-Aldrich (Shanghai, China). Biotin-labeled CL and PG were synthesized by Echelon Biosciences (Shanghai, China). SYTO™ 9 green fluorescent nucleic acid label (S34854; 5 mM) was purchased from Thermo Fisher (Shanghai, China).

### Antimicrobial susceptibility testing

Antimicrobial and AMXT-1501 MICs were determined by the broth macrodilution method in cation-adjusted Mueller-Hinton broth according to Clinical and Laboratory Standards Institute guidelines (CLSI-M100-S27). The range of concentrations (2-fold dilutions) tested for antimicrobials was 0.5–128 μg/mL, for AMXT-1501 was 1.56–50 μM (1.12–35.74 μg /mL). Strains grow without antimicrobial or AMXT-1501 were the control groups. Antimicrobial susceptibility results were confirmed based on CLSI-M100-S27. The ESBL production was detected by the Phoenix 100 automated microbiology system (BD) and determined by the double-disk synergy test using CTX and CAZ with and without clavulanic acid (BD, Franklin Lakes, UN) in accordance with CLSI-M100-S27. CRE was detected by the Phoenix 100 automated microbiology system (BD) and was confirmed by the determination of imipenem or meropenem MICs as prescribed in CLSI-M100-S27. All experiments were performed in triplicate.

### Checkerboard studies

FICs were determined with checkerboard assays [20]. CAMHB medium was dispensed into each well of a 96-well plate (100 μl/well). Antimicrobials were diluted along the abscissa whereas AMXT-1501 was diluted along the ordinate. The range of concentrations (2-fold dilutions) tested for antimicrobials or AMXT-1501 were as same as for antimicrobial susceptibility testing. Strains grown without antimicrobial or AMXT-1501 were the control groups. Overnight bacterial cultures were standardized to match a 0.5 McFarland and followed by 1:100 dilution in CAMHB. The MIC was recorded as the well with the lowest concentration of antimicrobial with no visible growth after incubation at 37 °C for 18 h. The FIC was calculated and synergy was defined as described in detail previously [20].

### Mammalian cell safety assessment

Haemolytic activity was determined on sheep cells. Briefly, sheep blood cells were prepared from fresh, sterile, defibrinated blood (Bersee, Beijing) treated with AMXT-1501 (1.56–100 μM) at 37°C for 1 h; 1.0% Triton X-100 and 0.9% NaCl were used as positive and negative controls, respectively. Absorption of released haemoglobin was measured at 570 nm.

Cytotoxicity assays were performed on J774 mouse monocytes, Huh7 human liver cancer cells, 293 T human renal epithelial cells, HUVECs, and A549 human lung cancer cells with a CCK-8 kit (MedChemExpress, Shanghai, China). AMXT-1501 (1.56–100 μM) and 1.25 × 10^4^ cells were added simultaneously to 96-well plate wells, cultured in Dulbecco’s modified Eagle medium supplemented with 1% heat-inactivated fetal bovine serum, 1% (w:v) penicillin–streptomycin, and 1% (w:v) sodium pyruvate (Sigma-Aldrich) at 37°C under 5% CO_2_ for 24 h, followed by CCK-8 assays. CCK-8 solution (10 μL/well) was incubated with the cultures for 1.5 h, and then absorbance was measured at 450 nm. Cell viabilities (%) were calculated as: (absorbance of the experimental group – absorbance of the blank group)/(absorbance of the control group – absorbance of the blank group) × 100%.

### Pharmacokinetic analysis

The pharmacokinetic characteristics of AMXT-1501 in vivo were detected according to a previous study [21]. Briefly, BALB/c female mice were intraperitoneally injected with a single dose of AMXT-1501 (20 mg/kg). Plasma samples were taken from three mice at each time point. Plasma was mixed with acetonitrile, vigorously vortexed, and centrifuged. The precipitate was re-extracted with acetonitrile, and supernatants were filtered through a 0.22-μm filter membrane. AMXT-1501 concentrations in supernatants were determined by AB SCIEX QTRAP 4500™ mass spectrometer (Applied Biosystems, CA, USA). Pharmacokinetic parameters were performed using a non-compartmental analysis model by WinNonlin 6.4 software.

### Time-kill assay

Time-kill assays were performed as described previously [22]. Overnight *S. aureus*, *E. coli*, and *K. pneumoniae* cultures were diluted 1:200 in fresh CAMHB and cultured to the mid-logarithmic growth phase (3.5 h) in 14-ml polypropylene round-bottom tubes (final volume, 6 ml). Vancomycin, meropenem, tigecycline and AMXT-1501 were added to cultures (final concentration at 8× MIC) and incubated at 37 °C with shaking. Strains grown without antimicrobial or AMXT-1501 were the control groups. At the time points of 0, 2, 6, 12, and 24 h, 1 ml of aliquots were sampled and washed with 0.9% saline solution. Ten-fold dilutions were plated on Muller-Hinton agar, and CFUs were counted. All experiments were performed in triplicate.

### Mice

Female 6–8-week BALB/c mice (18–22 g) were purchased from GemPharmatech (Jiangsu, China) and housed in a temperature-controlled room. Animal protocols were carried out in accordance with Care and Use of Laboratory Animals guidelines.

### Mice skin abscess model

BALB/c mice were anesthetized completely with 1% sodium pentobarbital (50 mg/kg body weight), and the hair on their back was removed. Then, 100 µl of *S. aureus* YUSA145 (MRSA)(1 × 10^7^ CFU) was inoculated subcutaneously in the back of mice, which were randomized into three groups (N = 15 per group). After 2 h of infection, mice in the treatment groups were injected intraperitoneally with vancomycin (20 mg/kg/body weight) or AMXT-1501 (20 mg/kg/body weight) every 12 h for 3 d. Mice injected with *S. aureus* YUSA145 (MRSA), but with no treatment served as the control group. The maximum length (L) and width (W) of the developing ulcer were measured on day 7. The lesion area was calculated as L × W. Lastly, the mice were sacrificed and their lesions were removed, weighed, homogenized in 500 μL of sterile phosphate-buffered saline (PBS), diluted 10-fold, and plated on tryptic soy agar. CFUs were counted after 48 h in culture at 37°C. Abscess bacteriology findings were expressed as means ± standard deviation (SD) log10 CFU/g.

### Mice abdominal infection model

BALB/c mice were completely anesthetized with 1% sodium pentobarbital (50 mg/kg body weight), then 100 µl of *E. coli* ECO2219 (CRE)(2 × 10^9^ CFU) was inoculated by intraperitoneal injection and randomly divided into three groups (N = 15 per group). After 2 h of infection, mice in the treatment groups were injected intraperitoneally with tigecycline (20 mg/kg/body weight) or AMXT-1501 (20 mg/kg/body weight) every 12 h for 3 d. Mice not injected with *E. coli* ECO2219 (CRE) served as the control group. Mice injected with *E. coli* ECO2219, but with no treatment served as the untreated group. The mortality rate of mice was recorded daily and the dead mice were collected. Lastly, all the living BALB/c mice were sacrificed on day 7, and with the previously collected dead mice, lungs and livers of those mice were removed, weighed, homogenized in 500 μL of sterile phosphate-buffered saline (PBS), diluted 10-fold, and plated on tryptic soy agar. CFUs were counted after 48 h in culture at 37°C. Lungs and livers bacteriology findings were expressed as means ± standard deviation (SD) log10 CFU/g.

### Growth curves

Overnight cultures of *S. aureus* and *E. faecalis* were diluted 1:200 in fresh TSB, combined with sub-MICs of AMXT-1501, and inoculated in 96-well polystyrene microtiter plates (300 μL/well); TSB without AMXT-1501 was used as untreated control. OD_600_ was determined by a Bioscreen C system (Lab Systems Helsinki, Finland). The experiment was recorded for 16 h. Each assay was performed in triplicate at least three times.

### Biofilm biomass determination

Biofilm biomasses of *S. aureus* and *E. faecalis* were determined by the crystal violet method [22]. To examine AMXT-1501 effects on biofilm formation, overnight cultures of *S. aureus* and *E. faecalis* were diluted 1:100 with fresh TSBG containing sub-MICs of AMXT-1501 inoculated into 96-well polystyrene microtiter plates; AMXT-1501 was omitted in controls. After static incubation for 24 h, supernatants were removed and plates were washed gently with PBS, dried at room temperature, and fixed in methanol for 15 min. Methanol was removed and cells were stained with 0.5% crystal violet for 10 min at room temperature. Crystal violet was dissolved in 95% ethanol and OD_570_ was determined. This experiment was performed in triplicate at least three times.

To examine AMXT-1501 effects on established biofilms, overnight cultures of *S. aureus* and *E. faecalis* were diluted 1:100 with fresh TSBG and inoculated into 96-well polystyrene microtiter plates. After static incubation for 24 h at 37°C (mature biofilms), supernatants were removed and plates were washed with 0.9% saline to remove unattached cells. Fresh TSBG containing AMXT-1501 was added, and no AMXT-1501 was used as a control condition. After static incubation for 24, 48 h, or 72 h, remaining biofilms were measured by the crystal violet method as described above. This experiment was performed in triplicate at least three times.

### SEM

Overnight *S. aureus* YUSA145 (MRSA) and *E. coli* ECO2219 (CRE) cultures were diluted 1:100 in TSB and cultured at 37°C for 4 h. AMXT-1501 (4× MIC) was added and the mixture was incubated at 37°C for 2 h. Strains grown without AMXT-1501 were the control group. Cells were collected by centrifugation, washed, ﬁxed in 2.5% glutaraldehyde for 2 h, washed again, and dehydrated in a series of ethanol solutions (50, 70, 80, 90, and 10% absolute ethanol, 15 min each). Coverslips were placed in a sample box, which was placed in a freeze-drying apparatus for 2 h. Prior to observation by SEM (EM-30AX microscope, COXEM), freeze-dried cultures were coated with gold spray for 3 min.

### TEM

TEM was performed as described previously [23]. AMXT-1501–treated *S. aureus* YUSA145 (MRSA) and *E. coli* ECO2219 (CRE) were collected by centrifugation (Strains grow without AMXT-1501 was the control group), washed, and ﬁxed overnight in 4% paraformaldehyde/0.1 M phosphate buffer at 4°C. After discarding the supernatants of fixed cells, 0.1 M phosphate buffer (pH 7.4) was added, and then the cells were re-suspended, washed, and dehydrated with an ethanol gradient (30, 50, 70, 80, 95, 100%, 20 min each). The dehydrated cells were treated with acetone and EMBed 812, followed by acetone and EMBed 812 (1:2) overnight at 37 °C, and then EMBed 812 for 6 h at 37°C. Embedding models with resin and samples were polymerized by heating to 65°C, cut to 60–80-nm thickness, and retrieved onto 50-mesh copper grids with formvar film. After staining with 2.6% lead citrate and three rinses with ultra-pure water, the copper grids were dried and placed into a grids board. The samples were observed under an HT 7800 microscope (Hitachi).

### Membrane integrity assay

*S. aureus* YUSA145 (MRSA) and *E. coli* ECO2219 (CRE) suspensions (OD_600 _= 0.2) were diluted 100-fold with PBS and pipetted into 24-well plates. AMXT-1501 or membrane phospholipids, SYTO-9, and PI solution were added to each well. Plates were kept at room temperature for 30 min and observed under a confocal laser scanning microscope (TCS SP8, Leica). A microplate reader was used to detect fluorescence intensity at excitation and emission wavelengths of 535 and 615 nm, respectively. Results were expressed in a relative fluorescence unit. All experiments were performed in triplicate.

### Membrane depolarization assay

*S. aureus* YUSA145 (MRSA) and *E. coli* ECO2219 (CRE) suspensions (OD_600 _= 0.2) were added into black, opaque, flat-bottomed 96-well plates, to which 1 μM DiBAC4(3) was added. The plates were then left for 30 min at 37°C in the dark. AMXT-1501 or membrane phospholipids were added, and fluorescence intensity was detected at 492 nm and 515 nm for 60 min. The results were expressed in a relative fluorescence unit. All experiments were performed in triplicate.

### ONPG hydrolysis assay

The inner membrane permeabilization of *E. coli* was detected by o-nitrophenyl-β-D-galactoside (ONPG) hydrolysis [24]. Briefly, *E. coli* ECO2219 (CRE) were grown to exponential phase, washed with an equal volume of buffer (10 mM Na_2_HPO_4_, 100 mM NaCl, pH 7.2) and diluted with 1.5 mM ONPG and AMXT-1501. The hydrolysis of ONPG to o-nitrophenol (ONP) over time was measured by the absorbance at 405 nm.

### Biolayer interferometry assay

AMXT-1501 binding affinities with CL and PG were determined with biolayer interferometry assays in a Gatorprime system (Gator Bio, San Francisco, USA) according to previous research [25]. After pre-wetting with kinetic buffer (PBS, 0.05% bovine serum albumin, 0.01% Tween 20), biotin-labeled CL or PG molecules were immobilized on streptavidin biosensor tips (Echelon Biosciences, Shanghai, China), which were loaded with AMXT-1501. Duplicate sensors (incubated in buffer without biotin-labeled CL or PG) were used as background binding controls. Interaction of AMXT-1501 with hydrophobic celastrol, hydrophobic chloromycetin with CL and PG were also measured as controls. The assays were conducted in 96-well black plates with a total volume of 300 μL/well at 30°C, following a standard protocol. Data analysis was performed in Gatorprime software, employing a double-reference subtraction protocol to account for non-specific and background signals, as well as signal drifts caused by biosensor variability. Equilibrium dissociation constant (Kd) values were calculated based on K_off_ to K_on_ ratios.

### Induction of AMXT-1501 non-sensitive clones in vitro

Inductions were performed as detailed previously [26]. *S. aureus, E. coli*, and *K. pneumoniae* strains were subcultured serially in TSB containing AMXT-1501 (initial concentration of 1/4× MIC, then increased successively), and were subcultured serially in TSB containing linezolid or tigecycline as control groups. Samples were cultured for 3∼5 passages before being exposed to the next concentration. Control samples were subcultured serially in plain TSB. Isolates from the last passage of each concentration were picked and cultured on tryptic soy agar plates without AMXT-1501 for two passages. Clones were selected and identified by matrix-assisted laser desorption ionization time-of-flight mass spectrometry (IVD MALDI Biotyper, Bruker, Bremen, Germany). MICs were re-determined and then the samples were stored at −80 °C in glycerol containing (35%) TSB.

### Whole-genome sequencing detection of mutations

Genomic DNA was extracted from *S. aureus* YUSA145 (MRSA) and *K. pneumoniae* NTUH-K2044 with a DNeasy Blood & Tissue Kit (Qiagen, Hilden, Germany). Sequencing libraries were prepared and whole genomes were sequenced in an Illumina HiSeq2500 sequencer. Gene functions were predicted by referring to the following databases: Gene Ontology, Kyoto Encyclopedia of Genes and Genomes, Clusters of Orthologous Groups, Non-Redundant Protein Database, Transporter Classification Database, and Swiss-Prot. Genomic alignments were performed with MUMmer and LASTZ tools. Single nucleotide polymorphisms, insertions, deletions, and structural variation annotations were identified based on inter-sample alignment results with MUMmer and LASTZ.

### Graphing and statistical analysis

Data were visualized in Prism 8.0 software (GraphPad Software, La Jolla, CA). Data were presented as means with SDs and were analyzed in SPSS v. 19.0 (Chicago, IL).

## Results

### Antibacterial activity against MDR bacteria

MedChemExpress Library chemicals were screened for activity against four reference bacteria strains: *S. aureus* ATCC29213, *S. aureus* SA113, *E. coli* ATCC25922, and *K. pneumoniae* NTUH-K2044. Each reference strain was inoculated, or not, with chemicals (50 μM) in 96-well polystyrene microtiter plates; after 24 h, the growth of planktonic cells in culture supernatants was measured. Chemicals that inhibited planktonic cell growth significantly, and whose anti-growth activity has not been described in the literature, were selected for further study.

The minimum inhibitory concentrations (MICs) of AMXT-1501 against clinical *S. aureus*, *Enterococcus faecium*, and *Enterococcus faecalis* isolates ranged from 3.13 μM (2.24 μg /mL) to 6.25 μM (4.47 μg /mL), with MIC_50_/MIC_90_ values of 6.25/6.25 μM (4.47 μg /mL)(Table 1). AMXT-1501 exhibited antibacterial activity against *Staphylococcus epidermids* [MIC_50_/MIC_90 _= 3.13/3.13 μM(2.24/2.24 μg /mL)], against ESBL-producing *E. coli* and *K. pneumoniae* [MIC_50_/MIC_90 _= 6.25/12.5 μM(4.47/8.93 μg /mL)], and against CR *E. coli*, *K. pneumoniae*, and *P. aeruginosa* [MIC_50_/MIC_90 _= 6.25–12.5 μM(4.47/8.93 μg /mL)](Table 1). AMXT-1501 did not suppress CR *A. baumanni* growth (Table 1).

**Table 1.** MICs of AMXT-1501 against Gram-positive and Gram-negative bacteria.

Strains (*n*)	AMXT-1501 MIC distributions (μM)						
	3.13	6.25	12.5	25	50	**>**50	MIC_50_/MIC_90_
**Gram-positive**							
MSSA (86)	27	59	-	-	-	-	6.25/6.25
MRSA (40)	3	37	-	-	-	-	6.25/6.25
Staphylococcus epidermids (32)	25	7	-	-	-	-	3.13/3.13
Enterococcus faecium (49)	4	45	-	-	-	-	6.25/6.25
Enterococcus faecalis (71)	5	66	-	-	-	-	6.25/6.25
**Gram-negative**							
ESBL-prod. *Escherichia coli* (184)	34	85	65	-	-	-	6.25/12.5
ESBL-prod. *Klebsiella pneumoniae* (162)	12	88	62	-	-	-	6.25/12.5
CR *Escherichia coli* (78)	-	-	69	9	-	-	12.5/12.5
CR *K. pneumoniae* (75)	-	-	65	10	-	-	12.5/12.5
CR *Acinetobacter baumanni* (36)	-	-	-	6	8	22	﹥50/﹥50
CR *Pseudomonas aeruginosa* (40)	1	22	17	-	-	-	6.25/12.5

CR, carbapenem-resistant; ESBL, extended-spectrum β-lactamase; MIC, minimum inhibitory concentration; MIC_50_/MIC_90_, the MIC values for 50% and 90% of bacterial growth inhibition; MRSA, methicillin-resistant *Staphylococcus aureus*; MSSA, methicillin-resistant *S. aureus*; prod., producing.

Concomitant exposure of MRSA to sub-MIC doses of AMXT-1501 with doxycycline resulted in antibacterial activity that was four times as potent as that seen with doxycycline alone (Table S1). However, AMXT-1501 did not have a potentiating effect on other antimicrobials against CR *E. coli* (Table S2).

### Safety and pharmacokinetic characteristics of AMXT-1501

*In vitro* toxicity assays showed that, at concentrations of ≤ 25 μM (17.87 μg /mL), AMXT-1501 had no haemolytic toxicity for human erythrocytes and only mild toxicity for five mammalian derived solid cells [J774 mouse monocytes, Huh7 human liver cancer cells, 293 T human renal epithelial cells, human umbilical vein endothelial cells (HUVECs), and A549 human lung cancer cells](Fig. S1). This low toxicity for mammalian cells at concentrations effective against MDR bacteria (reported above) makes AMXT-1501 suitable for testing for potential anti-infective applications.

Given the strong antimicrobial activity of AMXT-1501 and low cytotoxicity, we next conducted a pharmacokinetic (PK) study to evaluate the systemic exposure and blood residence time of AMXT-1501. A single dose of AMXT-1501 was administered to mice at up to 20 mg/kg intraperitoneally. Blood was collected at different times, and AMXT-1501 plasma levels were determined by LC-MS, and PK parameters were determined (Fig. S2).

### High comparative efficacy against MRSA and CR *E. coli*

**Figure 1.**
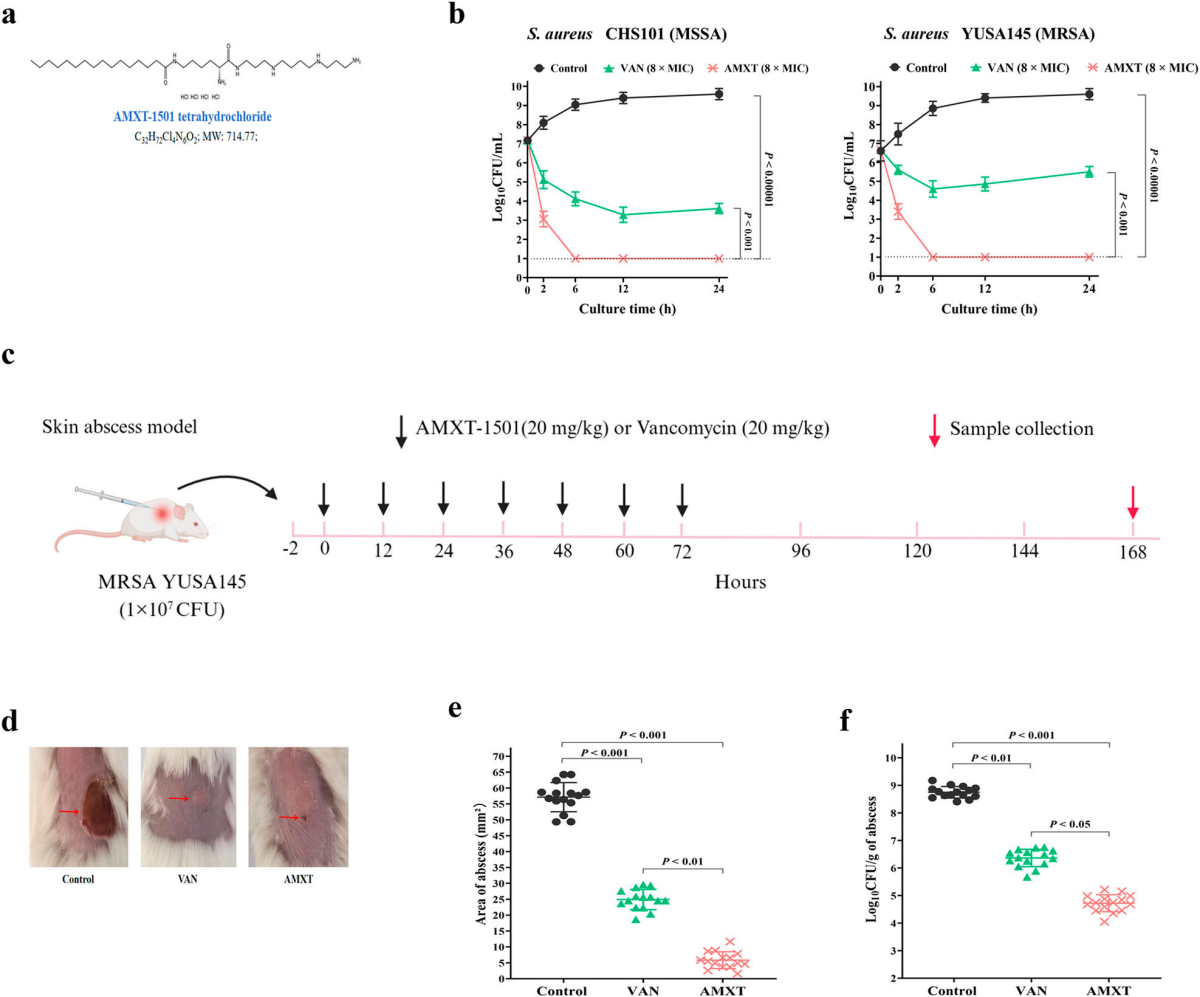
AMXT-1501 has excellent antibacterial activity against MDR *S. aureus*. (a) Molecular structure and formula of AMXT-1501 (AMXT). (b) Antibacterial activity of AMXT-1501 against MSSA CHS101 and MRSA YUSA145 detected by time-killing assays; compared with vancomycin (VAN); The dashed lines indicated the limit of detection of time-killing assays. Antibacterial activity of AMXT-1501 against skin abscess infected with MRSA YUSA145 (n = 15/group); compared with VAN. (c) The strategy of administration for mice skin abscess model, (d) representative abscesses, (e) lesion areas, and (f) bacteriology in abscesses on day 7. Data in panels b, e, and f were presented as means ± s.d, and *P* values were determined using an unpaired, two-tailed Student’s t-test. VAN, vancomycin; AMXT, AMXT-1501; MIC, minimum inhibitory concentration; MSSA, methicillin-sensitive *S. aureus*; MRSA, methicillin-resistant *S. aureus*;

**Figure 2.**
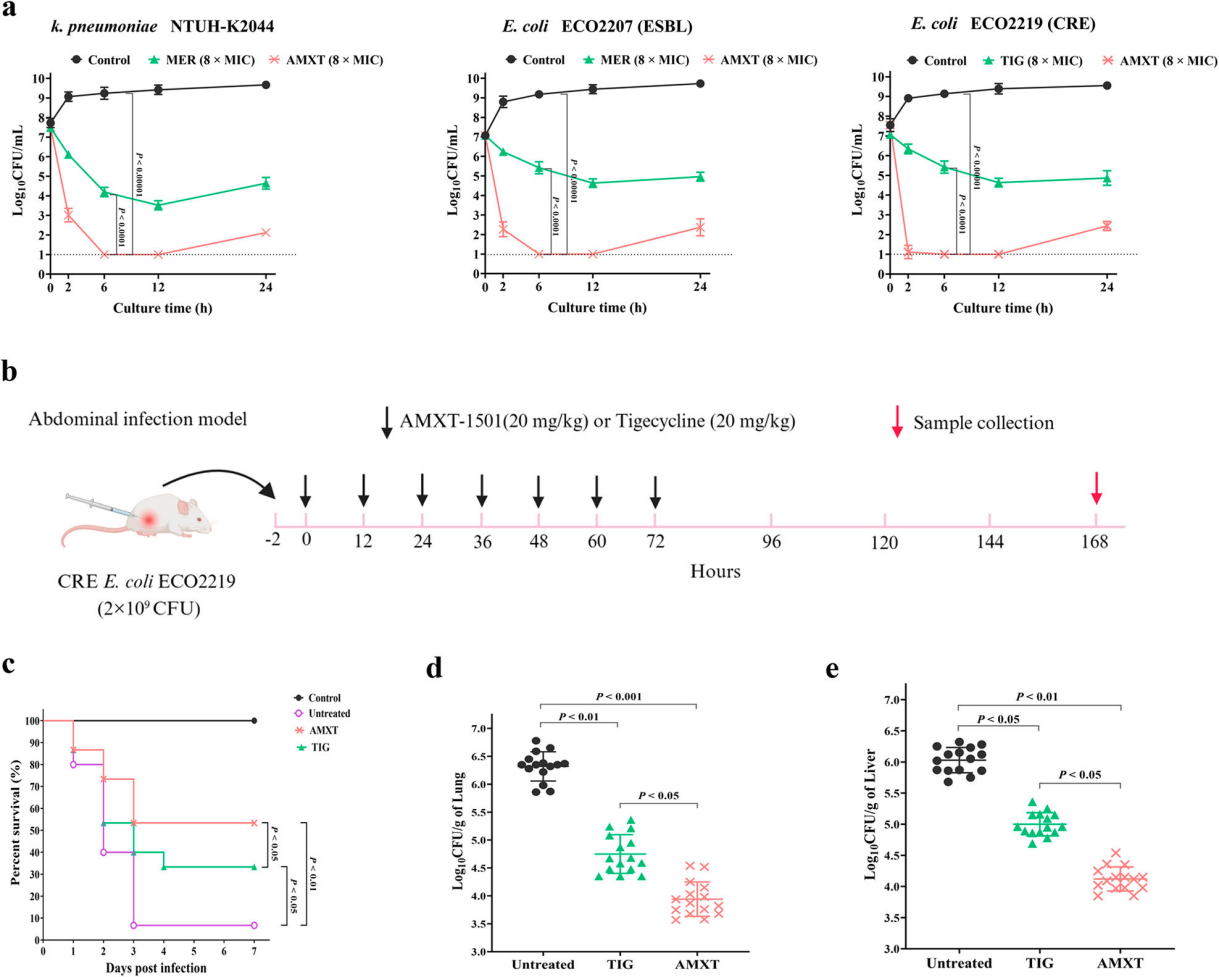
AMXT-1501 has excellent antibacterial activity against Gram-negative MDR bacteria. (a) Antibacterial activity of AMXT-1501 against *K. pneumoniae*, an ESBL-producing *E. coli*, and carbapenem-resistant Enterobacteriaceae (CRE) *E. coli* detected by time-killing assays; compared with meropenem (MER) or tigecycline (TIG); The dashed lines indicated the limit of detection of time-killing assays. Antibacterial activity of AMXT-1501 against abdominal infected with *E. coli* ECO2219 (*n* = 15/group); compared with TIG. (b) The strategy of administration for mice abdominal infection model, (c) survival rates, bacteriology in lung (d) and liver (e) on day 7. Data in panels a, d and e were presented as means ± s.d, and *P* values were determined using an unpaired, two-tailed Student’s t-test. *P* values were determined using log-rank test for panels c. MER, meropenem; TIG, tigecycline; AMXT, AMXT-1501; MIC, minimum inhibitory concentration; ESBL, extended-spectrum β-lactamase; CRE, carbapenem-resistant Enterobacteriaceae.

AMXT-1501 had rapid bactericidal effects on MRSA planktonic cells and killed more planktonic cells [≥3-log_10_ colony forming units (CFU)/ml] than vancomycin at 6 h, 12 h, and 24 h observations in time-kill assays (Figure 1(a,b)). In mice with subcutaneous abscess infections, abscess areas and intra-abscess bacterial loads were significantly smaller in mice treated with AMXT-1501 than in the vancomycin-treated group (Figure 1(c–f)). At the 6 h time-kill assay time point, AMXT-1501 had killed more planktonic ESBL-producing *E. coli* cells (≥4-log_10_ CFU/ml) than meropenem and had killed more CR *E. coli* cells than tigecycline (Figure 2(a)). In mice with abdominal infections, the survival rate in mice treated with AMXT-1501 was higher than that in the tigecycline-treated group, and bacterial loads in the lung and liver of the mice were also lower in the AMXT-1501-treated group than that in the tigecycline-treated group (Figure 2(b–e)).

### Reduction of *S. aureus* and *E. faecalis* biofilm formation

**Figure 3.**
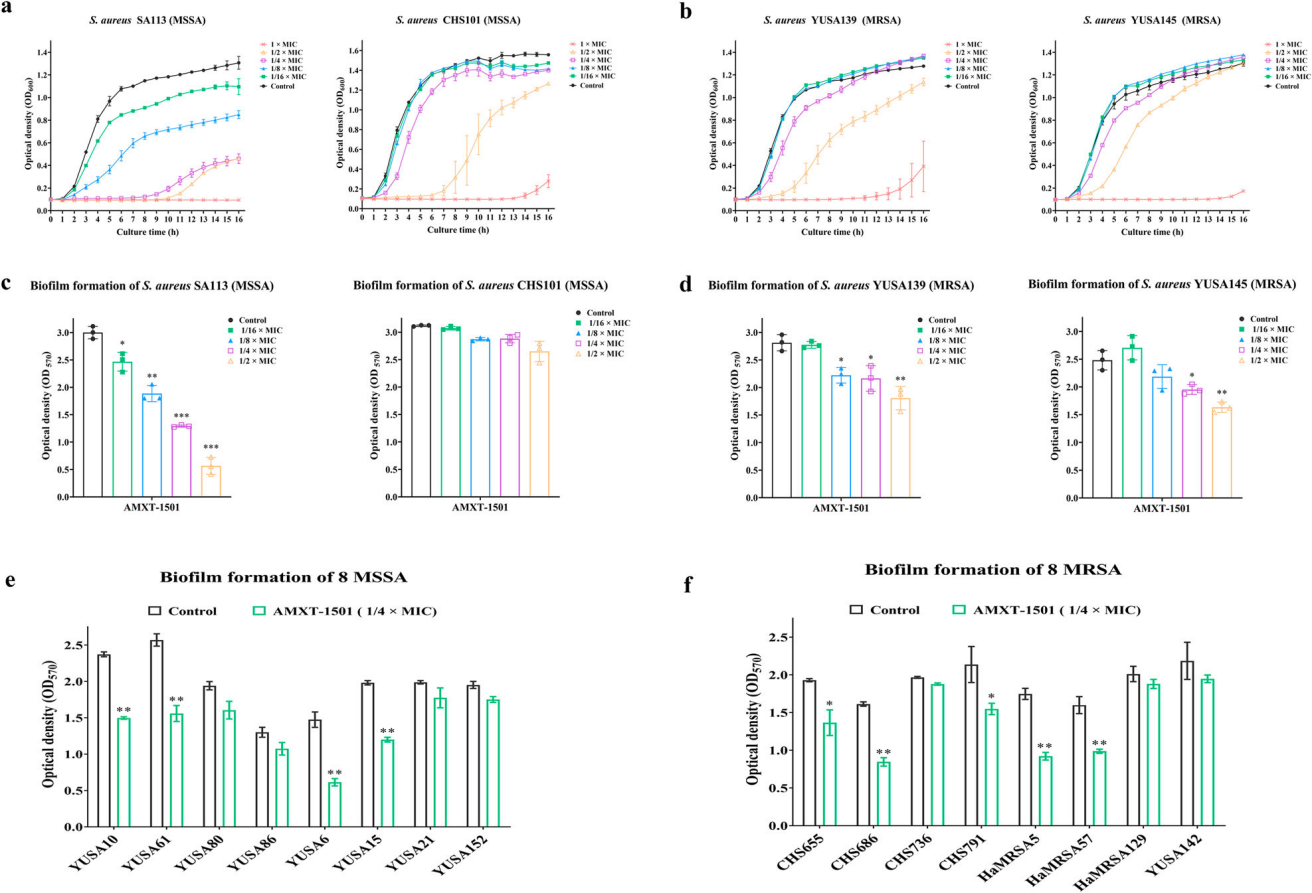
AMXT-1501 reduces the biofilm formation of *S. aureus*. Effects of sub-MICs of AMXT-1501 on growth of two MSSA (a, c) and two MRSA (b, d) isolates’ planktonic cells determined by optical density at 600 nm (OD_600_) and as indicated by biofilm biomass determination according to crystal violet staining analysis after 24 h of exposure. Effects of 1/4× MIC of AMXT-1501 on the biofilm formation of eight MSSA (e) and eight MRSA (f) strains determined by crystal violet staining analysis. Graphed data are means ± SDs; *P < 0.05, **P < 0.01. ***P < 0.001 vs. Control (unpaired, two-tailed Student’s t-test). MIC, minimum inhibitory concentration; MSSA, methicillin-sensitive *S. aureus*; MRSA, methicillin-resistant *S. aureus*;

To explore potential anti-biofilm AMXT-1501 effects, we first observed the effects of subinhibitory concentrations of AMXT-1501 on the growth of planktonic *S. aureus* and *E. faecalis* cells. At 1/2× MIC, AMXT-1501 inhibited growth for varying durations across strains, with growth of the methicillin-susceptible *S. aureus* (MSSA) strains SA113 and CHS101 being held down to negligible levels for ∼11 h and ∼ 7 h, respectively, the growth of both tested MRSA strains (YUSA139 and YUSA145) and of three of the four tested *E. faecalis* strains (16C30, 16C51, and 16C396) taking hold after only 4∼5 h, and the growth of a fourth *E. faecalis* strain (16C152) being uninhibited (Figure 3(a,b); Fig. S3a). At dosages at or below 1/4× MIC, AMXT-1501 was generally ineffective at suppressing the growth of these strains, with only SA113 exhibiting notable suppression in the presence of 1/4× MIC for ∼ 10 h (Figure 3(a,b); Fig. S3a).

Interestingly, OD_570_ (optical density at 570 nm) measurements indicated that at its 1/4× MIC, AMXT-1501 reduced *S. aureus* and *E. faecalis* biofilm formation substantially in seven of the above strains, with the exception being CHS101 (Figure 3(c, d); Fig. S3b). Examining biofilm formation in clinical isolates, we found that exposure to 1/4× MIC of AMXT-1501 reduced biofilm formation in four of eight MSSA isolate strains and in five of eight MRSA isolate strains (Figure 3(e,f)), but AMXT-1501 was totally ineffective against established *S. aureus* and *E. faecalis* biofilms (Fig. S4).

### AMXT-1501 destroys bacterial cell membranes

**Figure 4.**
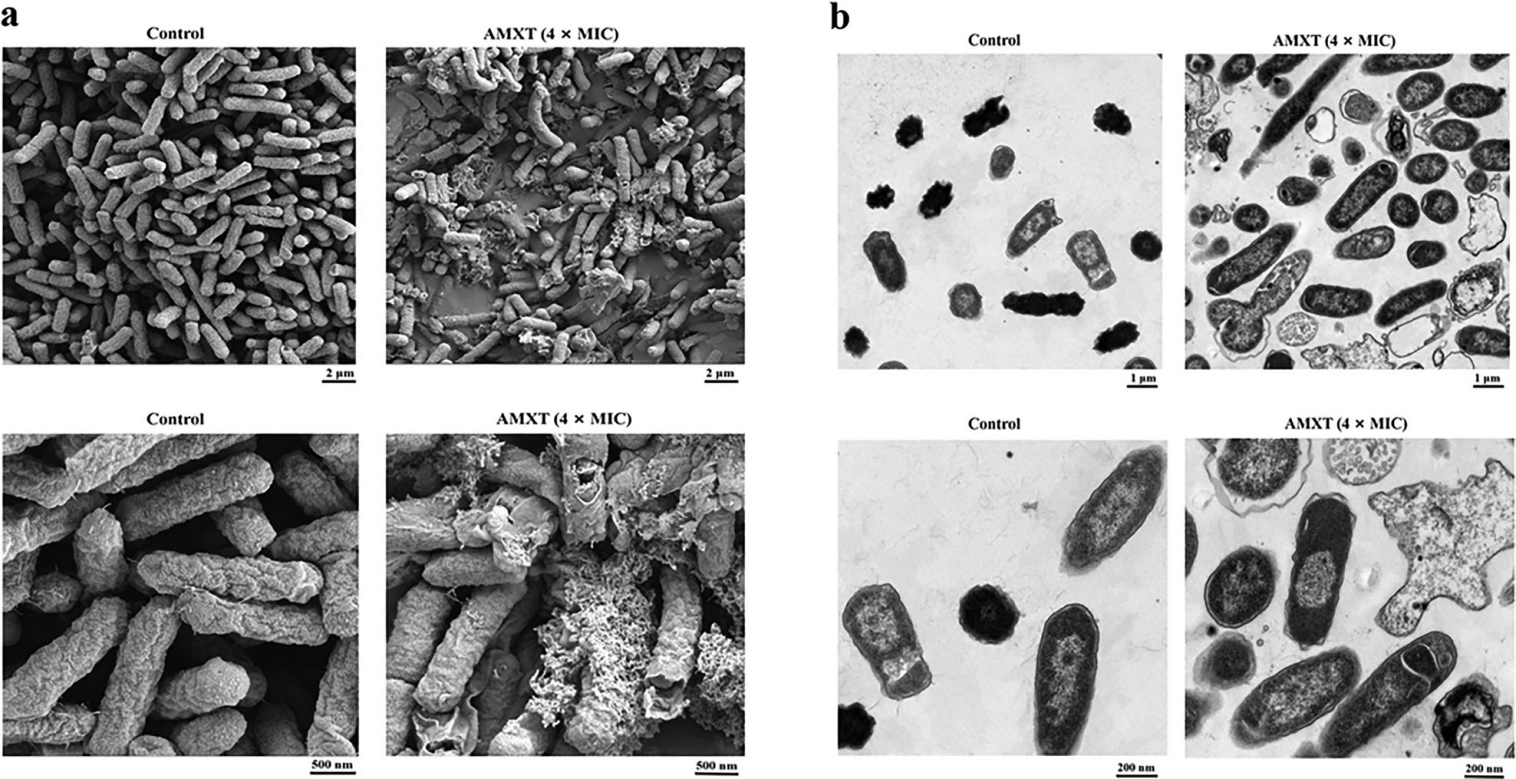
AMXT-1501 destroys bacterial cell membranes of *E. coli*. (a) *E. coli* ECO2219 (CRE) was treated with AMXT-1501 (AMXT) for 2 h and observed by SEM. (b) *E. coli* ECO2219 (CRE) was treated with AMXT-1501 for 2 h, and observed by TEM. Representative fields are shown in all panels. AMXT, AMXT-1501; MIC, minimum inhibitory concentration; CRE, carbapenem-resistant Enterobacteriaceae.

Scanning electron microscopy (SEM) studies were conducted to explore whether the broad-spectrum antibacterial activity of AMXT-1501 against MDR Gram-positive and -negative bacteria may be a consequence of effects on bacterial cell membranes. SEM analysis revealed that after a 2-h treatment with 4× MIC AMXT-1501, the cell membranes of *E. coli* ECO2219 (Figure 4(a)) and *S. aureus* YUSA145 (Fig. S5a) were damaged with effusion of intracellular substances, leading to a nonviable state. Transmission election microscopy (TEM) further confirmed damage to the cell membranes of *E. coli* ECO2219 (Figure 4(b)) and *S. aureus* YUSA145 (Fig. S5b) exposed to 4× MIC AMXT-1501.

**Figure 5.**
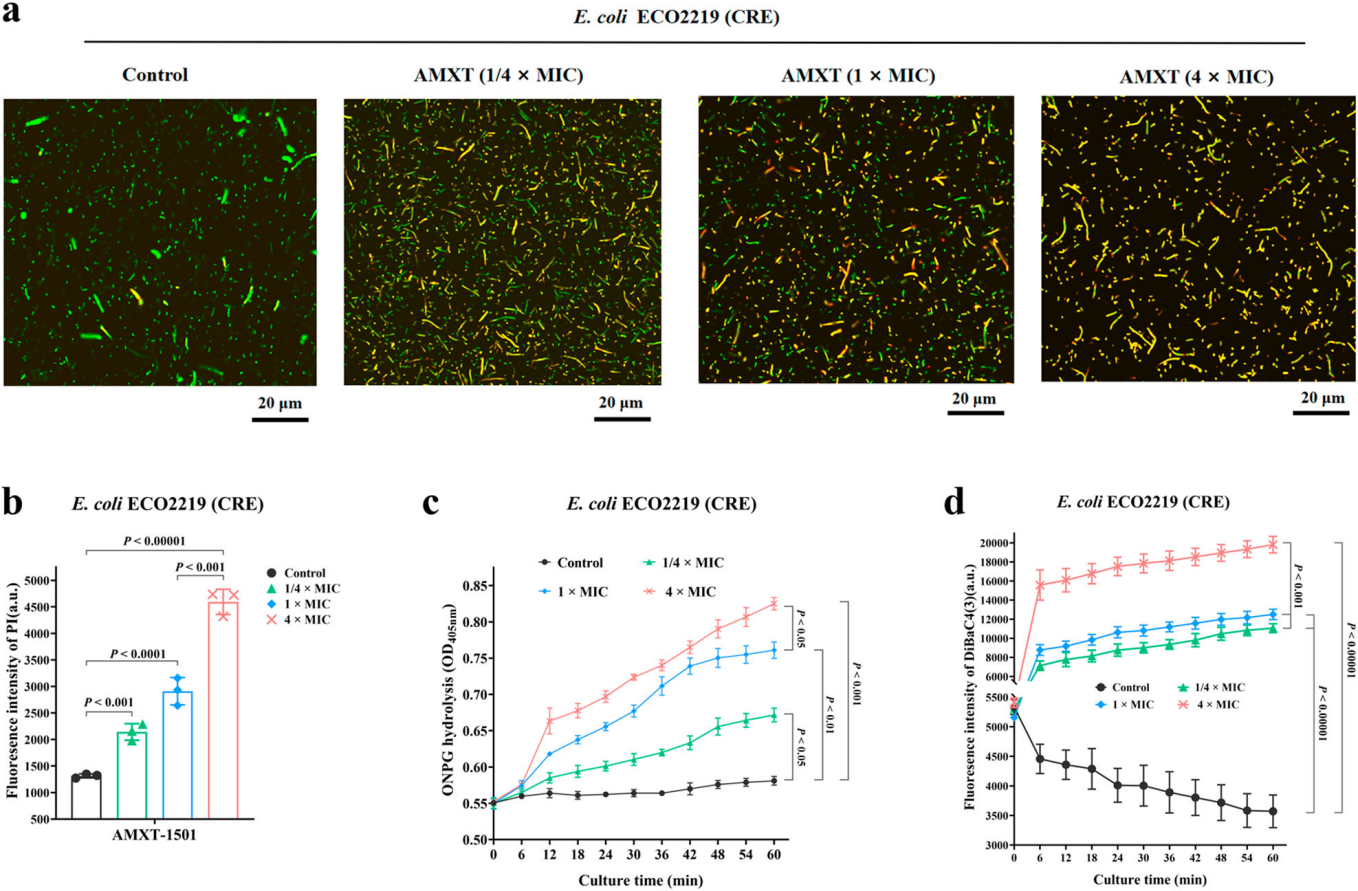
AMXT-1501 increased membrane permeability and depolarization of *E. coli*. (a) *E. coli* ECO2219 (CRE) treated with AMXT-1501 for 30 min, labelled with SYTO-9 and PI, and observed under a confocal laser scanning microscope; (b) Fluorescence intensities of PI-labelled *E. coli* ECO2219 (CRE) treated with AMXT-1501 for 30 min, detected at excitation and emission wavelengths of 535 and 615 nm measured by a microplate reader, respectively. (c) The inner membrane permeabilizing ability of AMXT-1501 was determined by measurement of β-galactosidase activity in *E. coli* ECO2219 (CRE) using the normally impermeable, chromogenic substrate o-nitrophenyl-β-d-galactoside (ONPG); (d) Fluorescence intensities of DiBAC4(3)-treated *E. coli* ECO2219 (CRE) treated with AMXT-1501, detected associated fluorescence intensity at 492 and 515 nm for 60 min, respectively. Representative fields are shown in panel a. Data in panels b, c and d were presented as means ± s.d, and *P* values were determined using an unpaired, two-tailed Student’s t-test. a.u., arbitrary units. AMXT, AMXT-1501; MIC, minimum inhibitory concentration; CRE, carbapenem-resistant Enterobacteriaceae.

Following exposure of *E. coli* ECO2219 to 4× MIC AMXT-1501, confocal laser scanning microscopy demonstrated cellular membrane permeability, evidenced by increased leakage of fluorescent nucleic acid dyes [SYTO-9 and propidium iodide (PI)] (Figure 5(a,b)) and ONPG (Figure 5(c)), as well as markedly increased membrane depolarization, evidenced by the voltage-sensitive fluorescent marker DiBaC4(3) [bis-(1,3-dibutylbarbituric acid) trimethine oxonol](Figure 5(d)). Similar results evidencing 4× MIC AMXT-1501–induced membrane permeability and depolarization were obtained in *S. aureus* YUSA145 (Fig. S6a-c).

### AMXT-1501 targets membrane phospholipids

**Figure 6.**
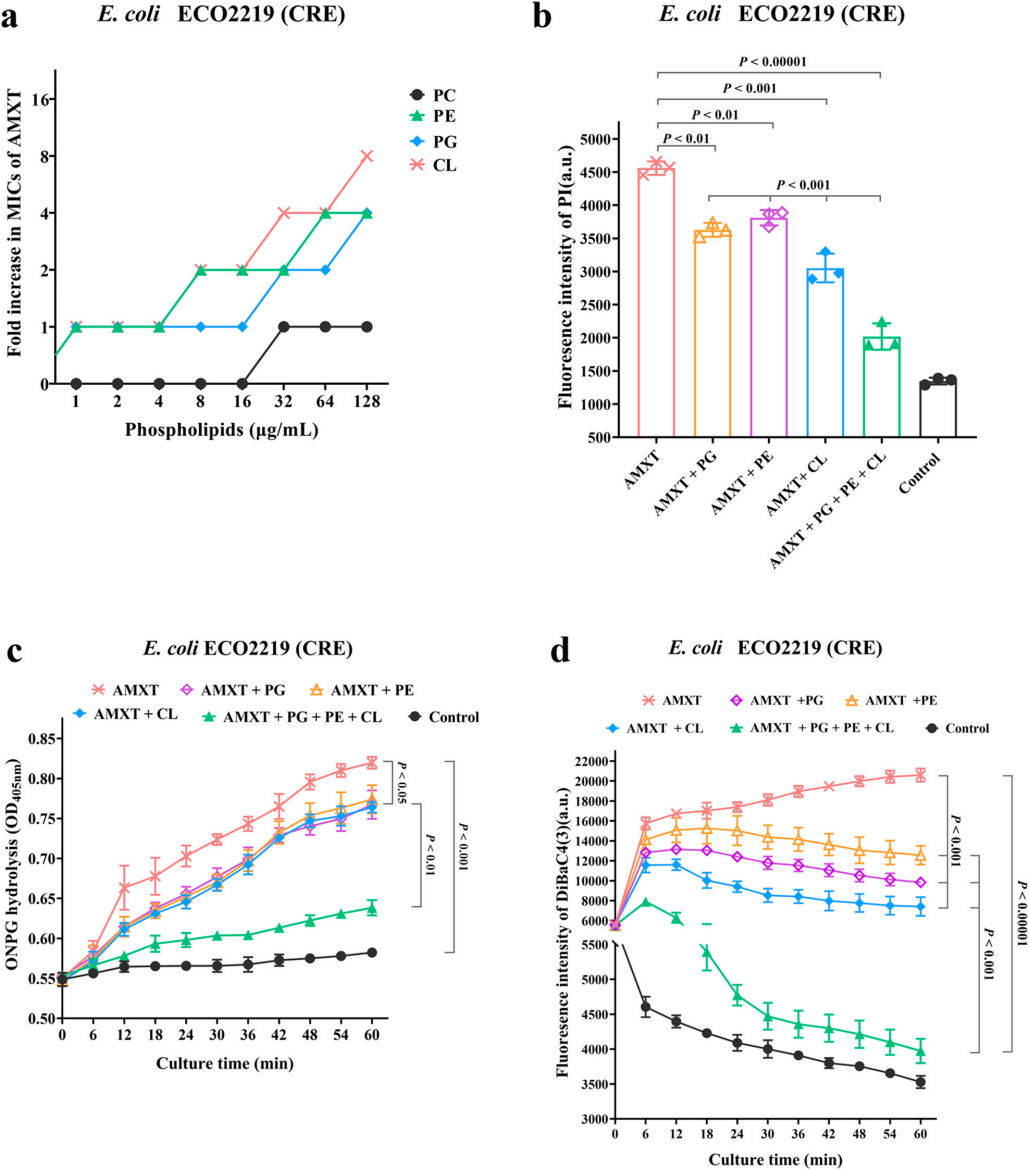
Bactericidal activity of AMXT-1501 via CL and PG against *E. coli*. (a) Increased MICs of AMXT-1501 against *E. coli* ECO2219 (CRE) in the presence of PC, PE, PG, and CL; exogenous addition of CL, PG and PE attenuated AMXT-1501 effects on membrane permeability (b, c) and depolarization (d). Data in panels b, c and d were presented as means ± s.d, and *P* values were determined using an unpaired, two-tailed Student’s t-test. a.u., arbitrary units. AMXT, AMXT-1501; MIC, minimum inhibitory concentration; CRE, carbapenem-resistant Enterobacteriaceae. PC, phosphatidylcholine; PE, phosphatidylethanolamine; PG, phosphatidylglycerol; CL, cardiolipin.

**Figure 7.**
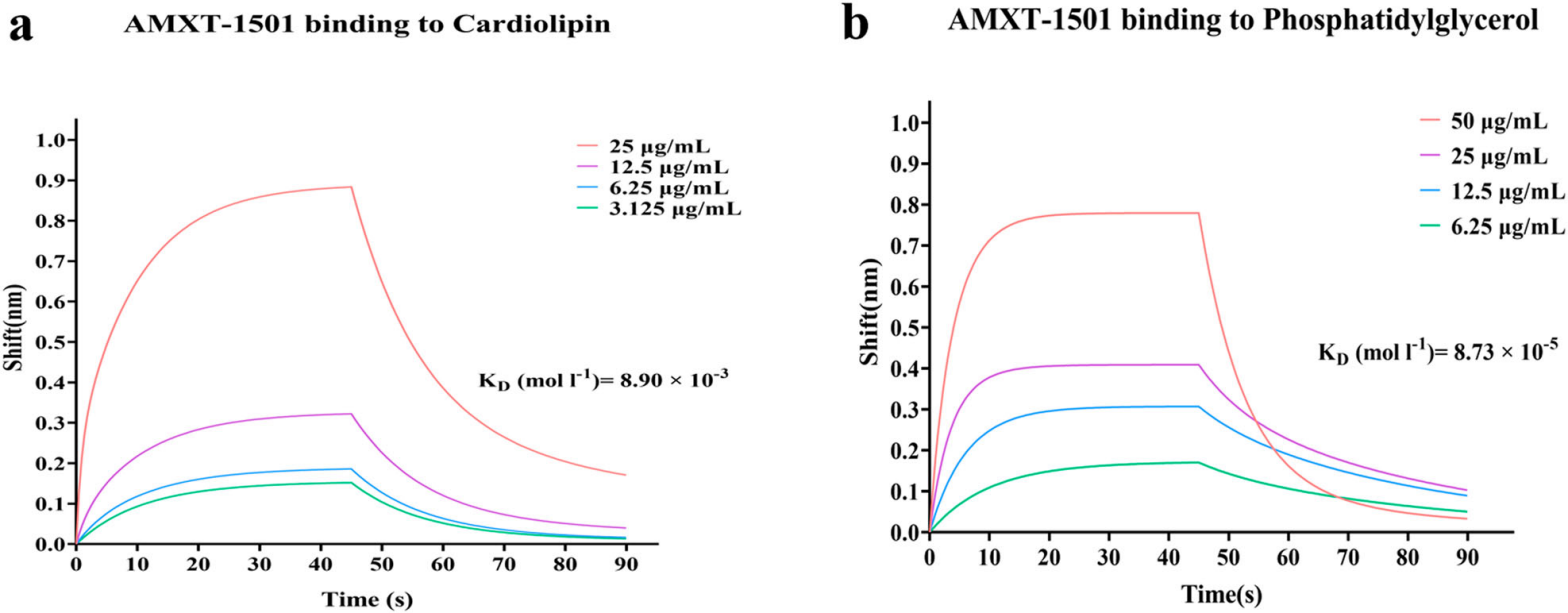
AMXT-1501 binding with CL and PG. Interaction of AMXT-1501 with CL (a) and with PG (b) measured by biolayer interferometry assay. CL, cardiolipin; PG, phosphatidylglycerol.

Fractional inhibitory concentrations (FICs) were determined to examine AMXT-1501 interactions with cell membrane constituents, including lipopolysaccharide (LPS) and membrane phospholipids [cardiolipin (CL); phosphatidylglycerol (PG); phosphatidylcholine (PC); and phosphatidylethanolamine (PE)] (Fig. S7). The FIC data indicated that CL, PG, and PE reduced the antibacterial efficacy of AMXT-1501 against *E. coli* ECO2219 in a dose-dependent manner (Figure 6(a)), and these effects were accompanied by associated attenuations in AMXT-1501 effects on cell membrane permeability and depolarization (Figure 6(b–d)). Meanwhile, CL and PG inhibited the antibacterial activity of AMXT-1501 against *S. aureus* YUSA145 in a dose-dependent manner, and these effects were also accompanied by associated attenuations in AMXT-1501 effects on cell membrane permeability and depolarization (Fig. S8a–c). Biolayer interferometry assays demonstrated that AMXT-1501 interacts strongly with CL and PG (Figure 7(a,b)). However, there were no binding interactions between AMXT-1501 with hydrophobic celastrol, between hydrophobic chloromycetin with CL or PG(Fig. S9).

Peptidoglycan, the main component of Gram-positive bacterial cell walls, did not affect the antibacterial activity of AMXT-1501 against *S. aureus* YUSA145 (Fig. S10a). LPS, the main component of Gram-negative bacterial cell membranes, did not affect the antibacterial activity of AMXT-1501 against *E. coli* ECO2219 (Fig. S10c). Bacterial polyamines (spermine, spermidine, 1,4-diaminobutane dihydrochloride, and 1,5-diaminopentane dihydrochloride) did not alter AMXT-1501 antibacterial activity against *S. aureus* YUSA145 (Fig. S10b) or *E. coli* ECO2219 (Fig. S10d).

### Genetic mutations in AMXT-1501–induced isolates

This study aims to explore resistant mutations of bacteria through continuous subculture under AMXT-1501 pressure, thus potential targets of AMXT-1501 can be revealed based on resistant mutations. Resistant mutations of bacteria were examined through continuous subculture under AMXT-1501 pressure. However, only one (of three) *S. aureus* strains, namely YUSA145, and only one Gram-negative bacterium (of three), namely *K. pneumoniae* NTUH-K2044, achieved 2-fold increases in AMXT-1501 MICs after 100 d of induction (Fig. S11 and S12). In control experiments, all six of the tested strains developed multi-fold increases in linezolid and tigecycline MICs after 100 d of induction (Fig. S13 and S14). Although our objective of exploring the potential targets of AMXT-1501 in bacteria by inducing AMXT-1501 resistant mutations has not been achieved, we have also found that AMXT-1501 has a relatively high resistance barrier, a potential advantage for anti-infection applications.

Finally, the *S. aureus* YUSA145 and *K. pneumoniae* NTUH-K2044 with the 2-fold increases in AMXT-1501 MICs were sequenced and found some gene mutations related to stress. AMXT-1501 induced six nonsynonymous amino-acid mutations in *S. aureus* YUSA145, including one each in AroH/AroA I beta, ArsR/SmtB family transcription factor, and cadmium resistance transporter CadD (Table S3). Meanwhile, AMXT-1501 induced >50 nonsynonymous amino acid mutations in *K. pneumoniae* NTUH-K2044, including 26 mutations in siderophore salmochelin receptor IroN, 4 in esterase family protein, 3 in ABC transporter ATP-binding protein, and 3 in ATP-binding cassette domain-containing protein (Table S4).

## Discussion

This study demonstrated for the first time that AMXT-1501 has antibacterial activity against Gram-positive and -negative MDR bacteria, including MRSA, ESBL-producing *E. coli*, *K. pneumoniae,* CR *E. coli*, *K. pneumoniae*, and *P. aeruginosa.* These results indicate that AMXT-1501 should be further explored as a potential antibacterial that may provide efficacy against difficult-to-eradicate MDR bacteria.

AMXT-1501 is a polyamine transport system inhibitor that blocks tumour growth in immunocompetent mice and diffuses intrinsic pontine glioma orthotopic animal models [19, 27]. It has been shown to act synergistically with difluoromethylornithine in multiple ways, including reducing neuroblastoma cell viability, blocking spermidine uptake in neuroblastoma cell lines, inhibiting polyamine synthesis and uptake, reducing tumour progression, and prolonging survival in mice with neuroblastomas [28]. Recently, AMXT-1501 was found to inhibit both polyamine and capsule biosynthesis in pneumococci in a serotype-dependent manner at physiologically relevant concentrations [29].

The MIC_50_/MIC_90_ values observed for AMXT-1501 against MRSA (6.25/6.25 μM, ∼4.47 μg/mL; Table S1) and against ESBL-producing and CR Enterobacteriaceae (6.25–12.5 μM, 4.47∼8.93 μg /mL; Table S2) in this study were approximately two times the MICs observed for vancomycin and tigecycline, respectively. However, the bactericidal activities of AMXT-1501 in *in vitro* time-kill assays and mouse infection models were superior to those of vancomycin and tigecycline. Moreover, under continuous AMXT-1501 pressure *in vitro*, these MDR strains did not develop resistance easily. These results indicate that AMXT-1501 has potential advantages in fighting Gram-positive and -negative MDR bacterial infections.

The present data showing broad-spectrum antibacterial activity of AMXT-1501 against MDR Gram-positive and -negative bacteria suggest that AMXT-1501 might manipulate a common mechanism across diverse bacterial species. This possibility is supported by our observations of AMXT-1501-induced bacterial cell membrane damage, increased membrane permeability, and increased membrane potentials. Song et al. reported that although SLAP-S25, a short linear antibacterial peptide, showed only weak antibacterial activity related to damaging cell membranes, it enhanced the efficacy of all major classes of antibiotics against MDR Gram-negative pathogens [20]. AMXT-1501 also disrupted bacterial cell membranes, but it enhanced the antibacterial activity of doxycycline against MRSA only slightly at subinhibitory concentrations.

Follow-up experiments seeking additional mechanistic information regarding AMXT-1501s effects on membranes showed that CL and PG were the main membrane-constituent targets of AMXT-1501 in both Gram-positive and Gram-negative MDR bacteria. Cell membrane phospholipids such as CL play a crucial role in bacterial physiology, particularly in maintaining cytoplasmic division and respiratory complexes [30]. CL accumulates at the cell poles and membranes of rod-shaped bacteria (*E. coli* and *P. aeruginosa*, etc.) to support spatial separation, recruitment, and the activities of membrane proteins [31], making it an attractive antibacterial target. Accordingly, El Khoury et al. have developed an amphiphilic aminoglycoside antibacterial drug (3’,6-dinonyl amine) that causes CL migration and aggregation, thereby increasing membrane permeability and leading to death in *P. aeruginosa* [32].

Though there are many CL-binding antimicrobials, antibacterial agents that target PG are rare. Several studies demonstrated that daptomycin binds PG-containing lipids in the presence of calcium affecting the thermodynamic behaviour and promoting lipid extraction on different model lipids [33, 34]. PG-binding of theaflavin and its analogues, for example, can reduce the fluidity of the cell membrane of *Bacillus coagulans* [35]. PG is a predominantly anionic phospholipid in the inner membrane of bacteria, which may explain its broad-spectrum adjuvant activity against Gram-negative bacteria. SLAP-S25 enhances the efficacy LPS- and PG-targeted antimicrobials [20]. AMXT-1501 is a small-molecule chemical with substantially distinct characteristics from SLAP-25, including differences in structure and charge, that may explain the inability of AMXT-1501 to enhance the efficacy of other antimicrobials.

## Conclusion

This study demonstrated that AMXT-1501 has antibacterial activity against MDR Gram-positive and -negative bacteria, and that it was more effective against MRSA and CR *E. coli* than vancomycin and tigecycline, respectively*.* Subinhibitory concentrations of AMXT-1501 reduced the biofilm formation of *S. aureus* and *E. faecalis.* This study further identified CL and PG as the main targets of AMXT-1501 against MDR Gram-positive and -negative bacteria. Our findings of AMXT-1501 with excellent antibacterial activity provide an alternative and assistance for the current severe situation of treating MDR bacteria. Meanwhile, this study also found the main target of AMXT-1501 in MDR, which can be beneficial for improving the structure of AMXT-1501 to further enhance its antibacterial activity against MDR, and for its broad application prospects in clinical MDR infection treatment in the future. However, this study did not continue to modify the structure of AMXT-1501 to enhance its antibacterial activity based on its main targets CL and PG, which is a limitation of this study.

## Supplementary Material

Supplementary_Information

## Data Availability

The raw whole-genome sequencing data was posted in the Sequence Read Archive (SRA) database under accession number PRJNA1007457 (http://www.ncbi.nlm.nih.gov/bioproject/1007457). All the other data supporting the findings of this study are available within the article and its supplementary information files and from the corresponding authors upon reasonable request. A reporting summary for this article is available as a Supplementary Information file.
