# Supplementary material for: AMXT-1501 targets membrane phospholipids against Gram-positive and -negative multidrug-resistant bacteria
Source: Emerg Microbes Infect. 2024 Feb 29;13(1):2321981. doi: 10.1080/22221751.2024.2321981 (PMC10906134; doi:10.1080/22221751.2024.2321981)
Supplement: Supplementary_Information [file TEMI_A_2321981_SM3663.pdf]

Supplementary tables and figures:

Table S1. Potency of AMXT-1501 in combination with different classes of antimicrobials against the MRSA strain *S. aureus* YUSA145.

**Antimicrobial (class)**	**MIC[a] (μg/mL)**	**MIC[b] with AMXT-1501 (μg/mL)**	**FIC index**	**Potentiation[c] (fold)**
Oxacillin (penicillin)	>128	>128	>1	0
Ampicillin (penicillin)	128	128	>1	0
Cefazolin (cephalosporin)	>128	>128	>1	0
Ceftriaxone (cephalosporin)	>128	>128	>1	0
Cefepime (cephalosporin)	>128	>128	>1	0
Imipenem (carbapenem)	128	128	>1	0
Meropenem (carbapenem)	128	128	>1	0
Levofloxacin (quinolone)	>128	>128	>1	0
Erythromycin (macrolide)	>128	>128	>1	0
Azithromycin (macrolide)	>128	>128	>1	0
Doxycycline (tetracycline)	8	2	0.75	4
Tigecycline (glycylcycline)	1	1	>1	0
Gentamicin (aminoglycoside)	>128	>128	>1	0
Linezolid (oxazolidinone)	8	8	>1	0
Daptomycin (cyclic lipopeptide)	4	4	>1	0
Colistin (polymyxin)	>128	>128	>1	0
Vancomycin (glycopeptide)	2	2	> 1	0

Note: [a,b]MICs of antimicrobials in the absence or presence of 3.13 μM of AMXT-1501, respectively. [c]Potentiation of antimicrobials in the presence of 3.13 μM (2.24 μg/mL; 1/2 × MIC) of AMXT-1501. MIC, minimum inhibitory concentration; MRSA, methicillin-resistant *Staphylococcus aureus*.

Table S2. Potency of AMXT-1501 in combination with different classes of antimicrobials against the CR Enterobacteriaceae *E. coli* ECO2219.

**Antimicrobial (drug class)**	**MIC[a] (μg/mL)**	**MIC[b] with AMXT-1501 (μg /mL)**	**FIC index**	**Potentiation[c] (fold)**
Oxacillin (penicillin)	>128	>128	>1	0
Ampicillin (penicillin)	>128	>128	>1	0
Cefazolin (cephalosporin)	>128	>128	>1	0
Ceftriaxone (cephalosporin)	>128	>128	>1	0
Cefepime (cephalosporin)	>128	>128	>1	0
Imipenem (carbapenem)	128	128	>1	0
Meropenem (carbapenem)	>128	>128	>1	0
Levofloxacin (quinolone)	>128	>128	>1	0
Erythromycin (macrolide)	>128	>128	>1	0
Azithromycin (macrolide)	>128	>128	>1	0
Doxycycline (tetracycline)	32	32	>1	0
Tigecycline (glycylcycline)	4	4	>1	0
Gentamicin (aminoglycoside)	16	16	>1	0
Linezolid (oxazolidinone)	>128	>128	>1	0
Daptomycin (cyclic lipopeptide)	>128	>128	>1	0
Colistin (polymyxin)	2	2	>1	0
Vancomycin (glycopeptide)	>128	>128	>1	0

Note: [a,b]MICs of antimicrobials in the absence or presence of 6.25 μM of AMXT-1501, respectively. [c]Potentiation of antimicrobials in the presence of 6.25 μM (4.47 μg /mL; 1/2 × MIC) of AMXT-1501. MIC, minimum inhibitory concentration; CR, Carbapenem-resistant.

Table S3. Mutations in AMXT-1501 non-sensitive clone of *S. aureus* YUSA145 strain detected by the whole-genome sequencing.

**Ref_gene_ID**	**Mutation**		**Function/Subject_description**
	**Nt**	**AA**	
CYUSA145_GM001373	A808C	T270P	Bifunctional 3-deoxy-7-phosphoheptulonate synthase/chorismate mutase, AroH/AroA I beta
CYUSA145_GM002593	T16C	T6A	Metalloregulator ArsR/SmtB family transcription factor
CYUSA145_GM002594	T541C	I181V	Cadmium resistance transporter CadD
CYUSA145_GM002594	T499C	I167V	Hypothetical protein
CYUSA145_GM002594	C484A	A162S	Hypothetical protein
CYUSA145_GM002594	G424T	L142I	Hypothetical protein

Note: Bacteria were subcultured serially in TSB containing AMXT-1501. T1 clone from 35 YUSA145 generations (cultured for ~110 d) were subjected to whole-genome sequencing. AA, amino acid; Nt, nucleotide.

Table S4. Mutations in AMXT-1501 non-sensitive clone of *k. pneumoniae* NTUH-K2044 strain detected by whole-genome sequencing.

Ref_gene_ID	Mutation		Function/Subject_description
	Nt	AA	
MAZ46_002869	G78T	S26R	Two-component system sensor histidine kinase EnvZ
MAZ46_003226	A1631G	L544P	Endopeptidase La
MAZ46_004143	G879C	Y293X	Polysaccharide biosynthesis tyrosine autokinase
MAZ46_004346	A170C	D57A	AbrB/MazE/SpoVT family DNA-binding DCP
MAZ46_004346	T185C	V62A	AbrB/MazE/SpoVT family DNA-binding DCP
MAZ46_004347	G92T	G31V	Type II toxin-antitoxin system VapC family toxin
MAZ46_004347	A176G	H59R	Type II toxin-antitoxin system VapC family toxin
MAZ46_004349	C1846T	V616I	Siderophore salmochelin receptor IroN
MAZ46_004349	T1690C	T564A	Siderophore salmochelin receptor IroN
MAZ46_004349	A1667G	V556A	Siderophore salmochelin receptor IroN
MAZ46_004349	A1651C	L551V	Siderophore salmochelin receptor IroN
MAZ46_004349	G1606A	H536Y	Siderophore salmochelin receptor IroN
MAZ46_004349	T1543C	N515D	Siderophore salmochelin receptor IroN
MAZ46_004349	A1532T	I511N	Siderophore salmochelin receptor IroN
MAZ46_004349	C1462T	E488K	Siderophore salmochelin receptor IroN
MAZ46_004349	G1318A	P440S	Siderophore salmochelin receptor IroN
MAZ46_004349	T1295G	Q432P	Siderophore salmochelin receptor IroN
MAZ46_004349	T1281A	E427D	Siderophore salmochelin receptor IroN
MAZ46_004349	C1195T	D399N	Siderophore salmochelin receptor IroN
MAZ46_004349	T1169G	D390A	Siderophore salmochelin receptor IroN
MAZ46_004349	C1159T	E387K	Siderophore salmochelin receptor IroN
MAZ46_004349	G1142A	A381V	Siderophore salmochelin receptor IroN
MAZ46_004349	A1115G	L372P	Siderophore salmochelin receptor IroN
MAZ46_004349	G1112T	T371K	Siderophore salmochelin receptor IroN
MAZ46_004349	T1111C	T371A	Siderophore salmochelin receptor IroN
MAZ46_004349	G1093T	L365I	Siderophore salmochelin receptor IroN
MAZ46_004349	A1076C	L359W	Siderophore salmochelin receptor IroN
MAZ46_004349	T1072A	T358S	Siderophore salmochelin receptor IroN
MAZ46_004349	C1021T	G341S	Siderophore salmochelin receptor IroN
MAZ46_004349	G992T	T331N	Siderophore salmochelin receptor IroN
MAZ46_004349	T925C	T309A	Siderophore salmochelin receptor IroN
MAZ46_004349	C886T	E296K	Siderophore salmochelin receptor IroN
MAZ46_004349	C883G	G295R	Siderophore salmochelin receptor IroN
MAZ46_004349	C869T	S290N	Siderophore salmochelin receptor IroN
MAZ46_004349	C638T	R213H	Siderophore salmochelin receptor IroN
MAZ46_004349	C589T	A197T	Siderophore salmochelin receptor IroN

MAZ46_004349	C584T	R195H	Siderophore salmochelin receptor IroN
MAZ46_004350	C493A	L165I	Glycosyltransferase
MAZ46_004351	G713A	R238H	ABC transporter ATP-binding protein
MAZ46_004351	G822A	M274I	ABC transporter ATP-binding protein
MAZ46_004351	G1221T	Q407H	ABC transporter ATP-binding protein
MAZ46_004432	T265G	S89A	Transketolase
MAZ46_004432	C266A	S89Y	Transketolase
MAZ46_004567	C653T	R218Q	IS3 family transposase
MAZ46_004837	G266A	G89E	Esterase family protein
MAZ46_004837	G328A	V110I	Esterase family protein
MAZ46_004837	T359G	M120R	Esterase family protein
MAZ46_004837	C674T	A225V	Esterase family protein
MAZ46_005117	G1176C	X392Y	DUF4236 DCP
MAZ46_005344	G1286A	C429Y	ATP-binding cassette DCP
MAZ46_005344	T1295A	V432E	ATP-binding cassette DCP
MAZ46_005344	G356T	X119L	ATP-binding cassette DCP
MAZ46_005352	T34A	T12S	IS5/IS1182 family transposase

Note: AA, amino acid; Nt, nucleotide; DCP, domain-containing protein.

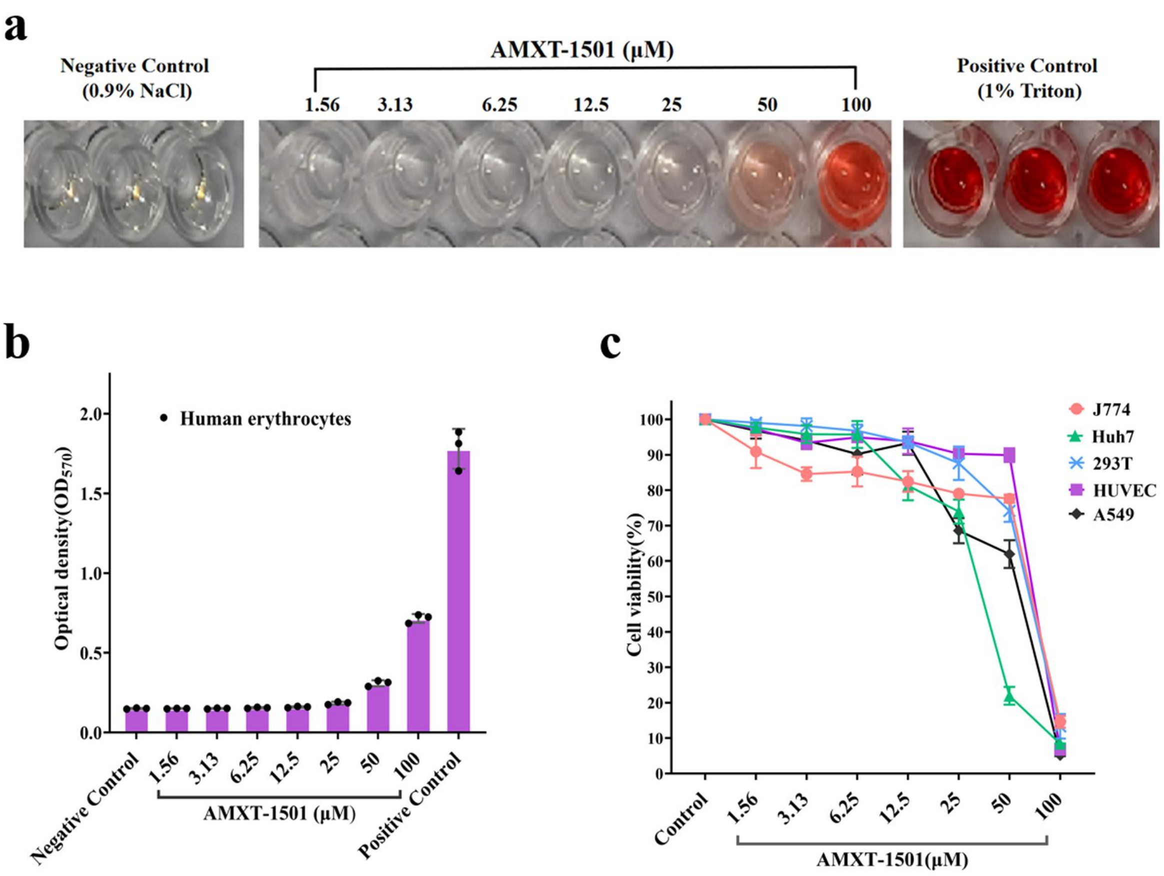

Figure S1. Safety of AMXT-1501 for mammalian cells. Assessment of AMXT-1501 toxicity for human erythrocytes (**a**), with associated OD measurements at 570 nm (**b**). (**c**) AMXT-1501 toxicity for J774 mouse monocyte macrophages, Huh7 human liver cancer cells, 293T human renal epithelial cells, HUVECs, and A549 human lung cancer cells were determined with CCK-8 assays. Representative fields are shown in panel **a**. Data in panel **b** and **c** are means with SDs.

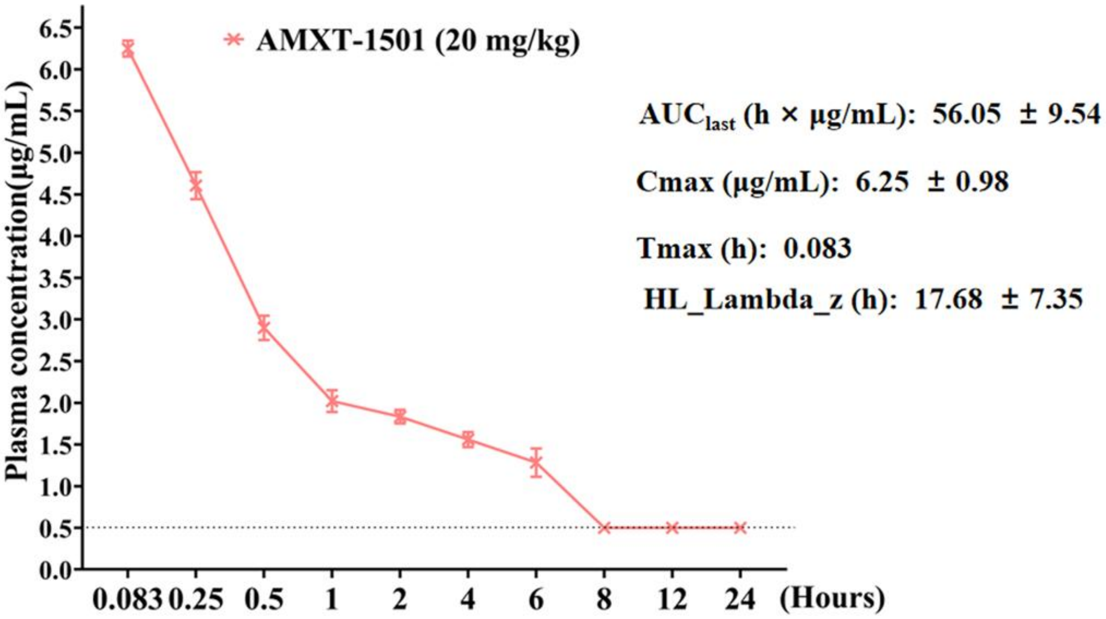

Figure S2. Pharmacokinetic analysis of AMXT-1501 in mice. The mean plasma concentrations of AMXT-1501 after a single *i.p.* injection of 20 mg/kg (3 mice per time point). All data are presented as mean ± SD. AUC_{last}: area under the plasma concentration-time curve from time 0 to 24 h; Cmax: maximal plasma concentration; Tmax: time to maximal plasma concentration; HL_Lambda_z: terminal half-life of the drug; The dashed line indicated the the limit of detection of LC-MS.

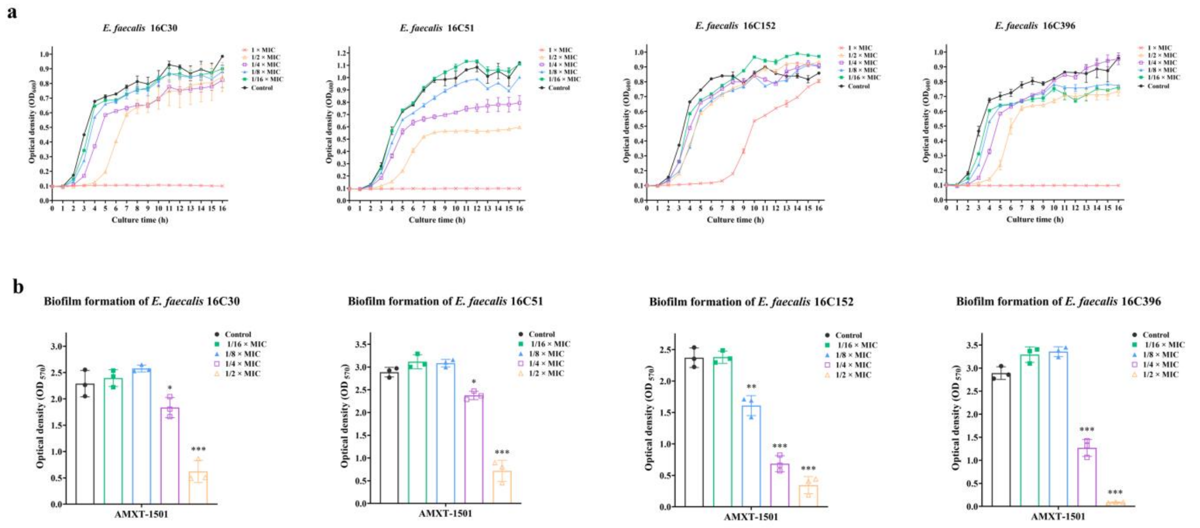

Figure S3. AMXT-1501 reduces the biofilm formation of *E. faecalis*. Effects of sub-MICs of AMXT-1501 on growth of four *E. faecalis* isolates' planktonic cells determined by optical density at 600 nm (OD_{600})(**a**) and as indicated by biofilm biomass determination according to crystal violet staining analysis after 24 h of exposure (**b**). Graphed data are means ± SDs; **P* < 0.05, ***P* < 0.01. ****P* < 0.001 vs. Control (unpaired, two-tailed Student's t-test). MIC, minimum inhibitory concentration;

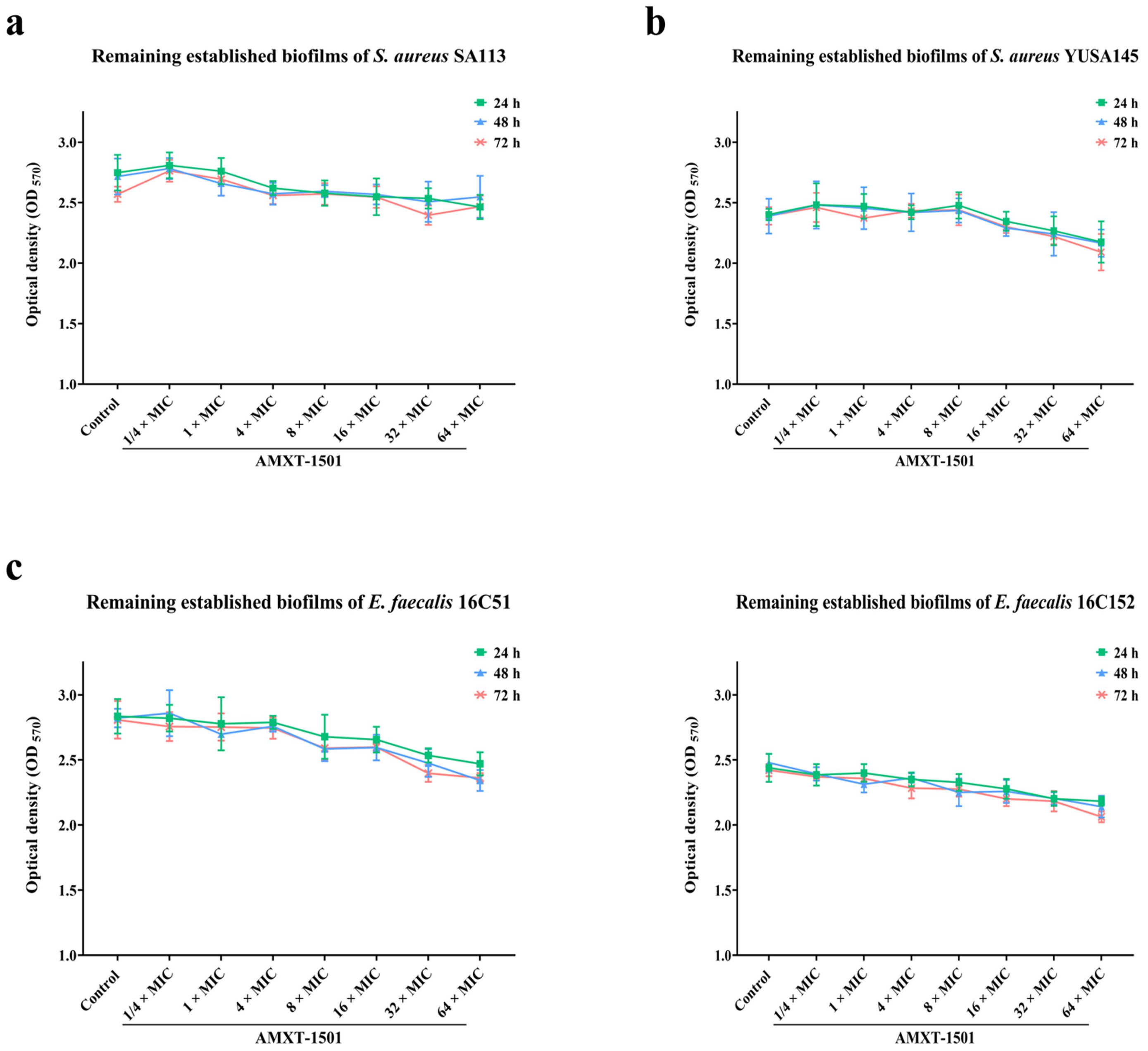

Figure S4. Effects of different concentrations of AMXT-1501 on established biofilms of *S. aureus* and *E. faecalis*. MSSA strain SA113 (**a**), MRSA strain YUSA145 (**b**), and two *E. faecalis* (**c**) isolates that had formed mature biofilms for 24 h were treated with AMXT-1501 at indicated concentrations, shown relative to MIC values for 24 h, 48 h or 72 h. Remaining biofilm biomasses were determined by crystal violet stain analysis. Data in all panels were presented as means ± s.d.

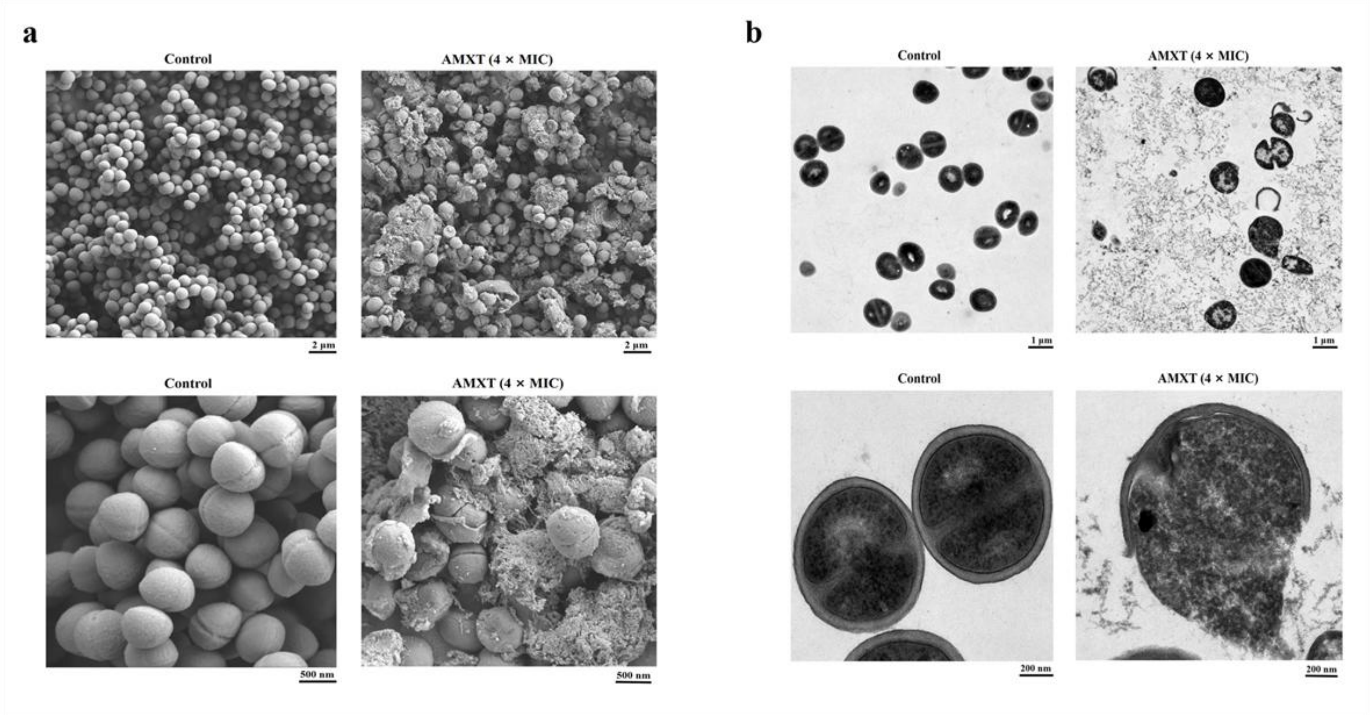

Figure S5. AMXT-1501 destroys bacterial cell membranes of *S. aureus*. (**a**) *S. aureus* YUSA145 (MRSA) was treated with AMXT-1501 for 2 h and observed by SEM. (**b**) *S. aureus* YUSA145 (MRSA) was treated with AMXT-1501 for 2 h, and observed by TEM. Representative fields are shown in all panels. AMXT, AMXT-1501; MIC, minimum inhibitory concentration; MRSA, methicillin-resistant *S. aureus*;

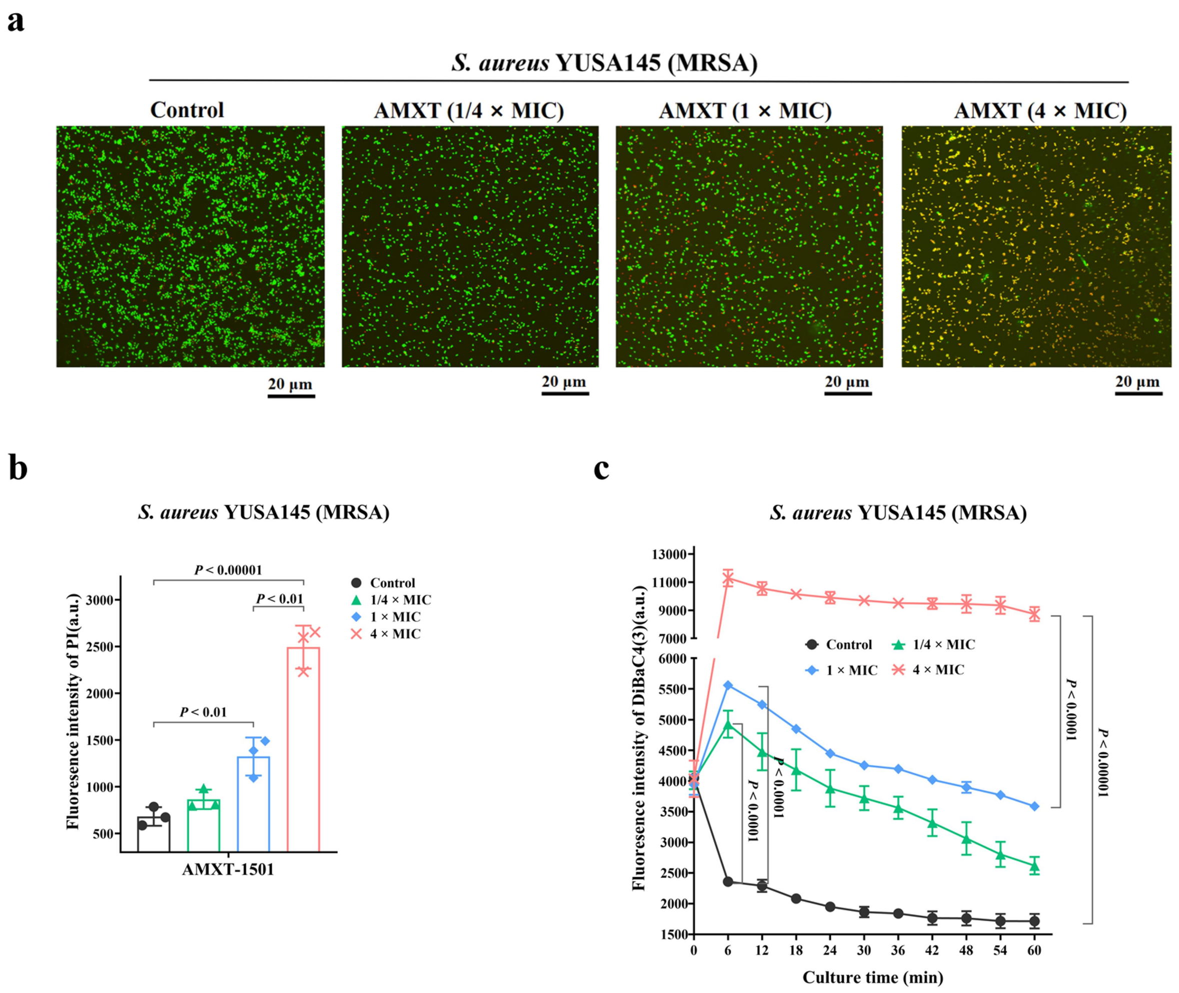

Figure S6. AMXT-1501 increased membrane permeability and depolarization of *S. aureus*. (**a**) *S. aureus* YUSA145 (MRSA) treated with AMXT-1501 for 30 min, labelled with SYTO-9 and PI, and observed under a confocal laser scanning microscope; (**b**) associated fluorescence intensity at excitation and emission wavelengths of 535 nm and 615 nm detected by a microplate reader, respectively. (**c**) *S. aureus* YUSA145 (MRSA) treated with AMXT-1501, stained with DiBAC4(3); associated fluorescence intensity at 492 nm and 515 nm for 60 min, respectively. Representative fields are shown in panel **a**. Data in panels **b** and **c** were presented as means ± s.d, and *P* values were determined using an unpaired, two-tailed Student's t-test. a.u., arbitrary units. AMXT, AMXT-1501; MIC, minimum inhibitory concentration; MRSA, methicillin-resistant *S. aureus*.

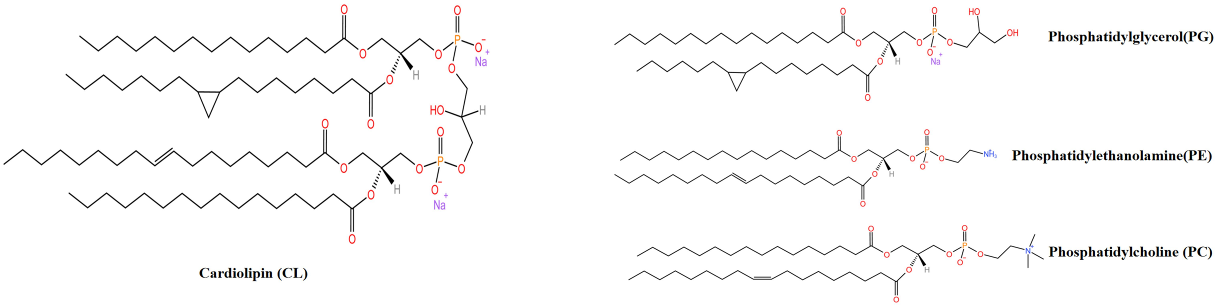

Figure S7. Molecular structure and formula of membrane phospholipids.

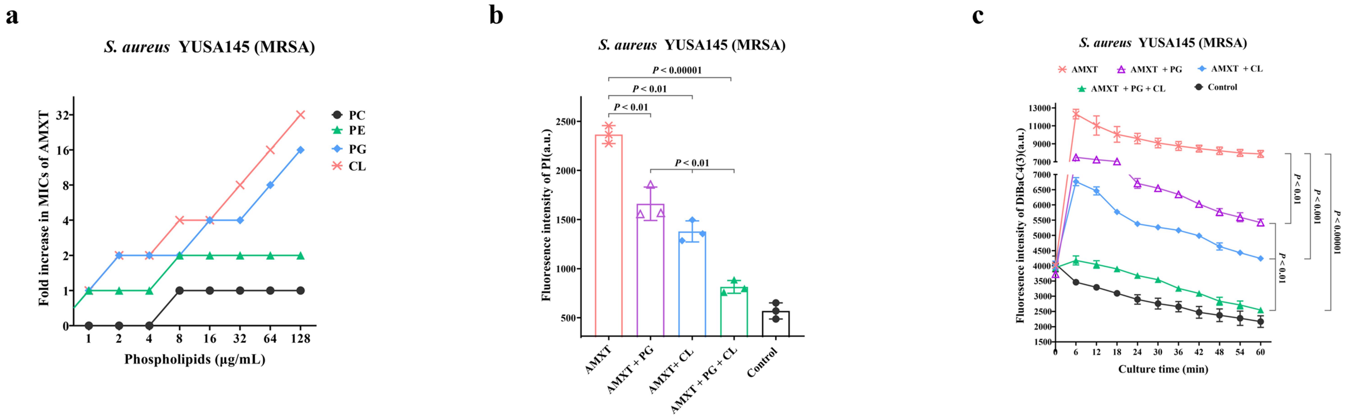

Figure S8. Bactericidal activity of AMXT-1501 via CL and PG against *S. aureus*. (a) Increased MICs of AMXT-1501 against *S. aureus* YUSA145 (MRSA) in the presence of PC, PE, PG, and CL; exogenous CL and PG attenuated AMXT-1501 effects on membrane permeability **(b)** and depolarization **(c)**. Data in panels **b** and **c** were presented as means ± s.d, and *P* values were determined using an unpaired, two-tailed Student's t-test. a.u., arbitrary units. AMXT, AMXT-1501; MIC, minimum inhibitory concentration; MRSA, methicillin-resistant *S. aureus*. PC, phosphatidylcholine; PE, phosphatidylethanolamine; PG, phosphatidylglycerol; CL, cardiolipin.

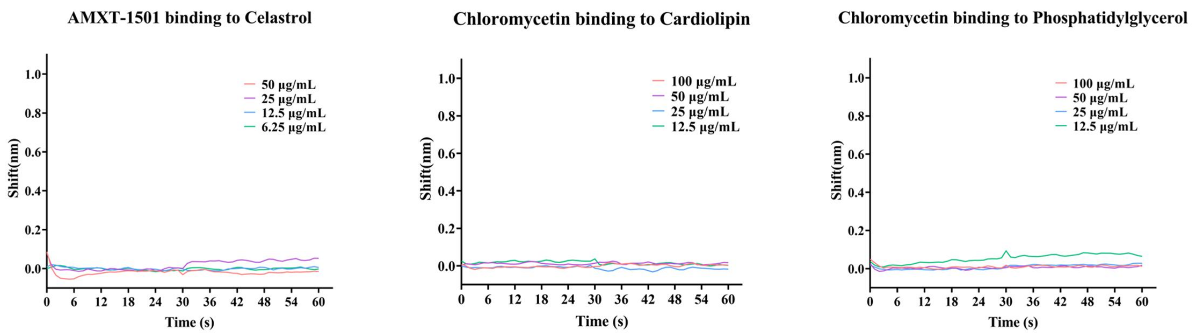

Figure S9. Interaction of AMXT-1501 with celastrol, chloromycetin with cardiolipin (CL) and phosphatidylglycerol (PG) measured by biolayer interferometry assay.

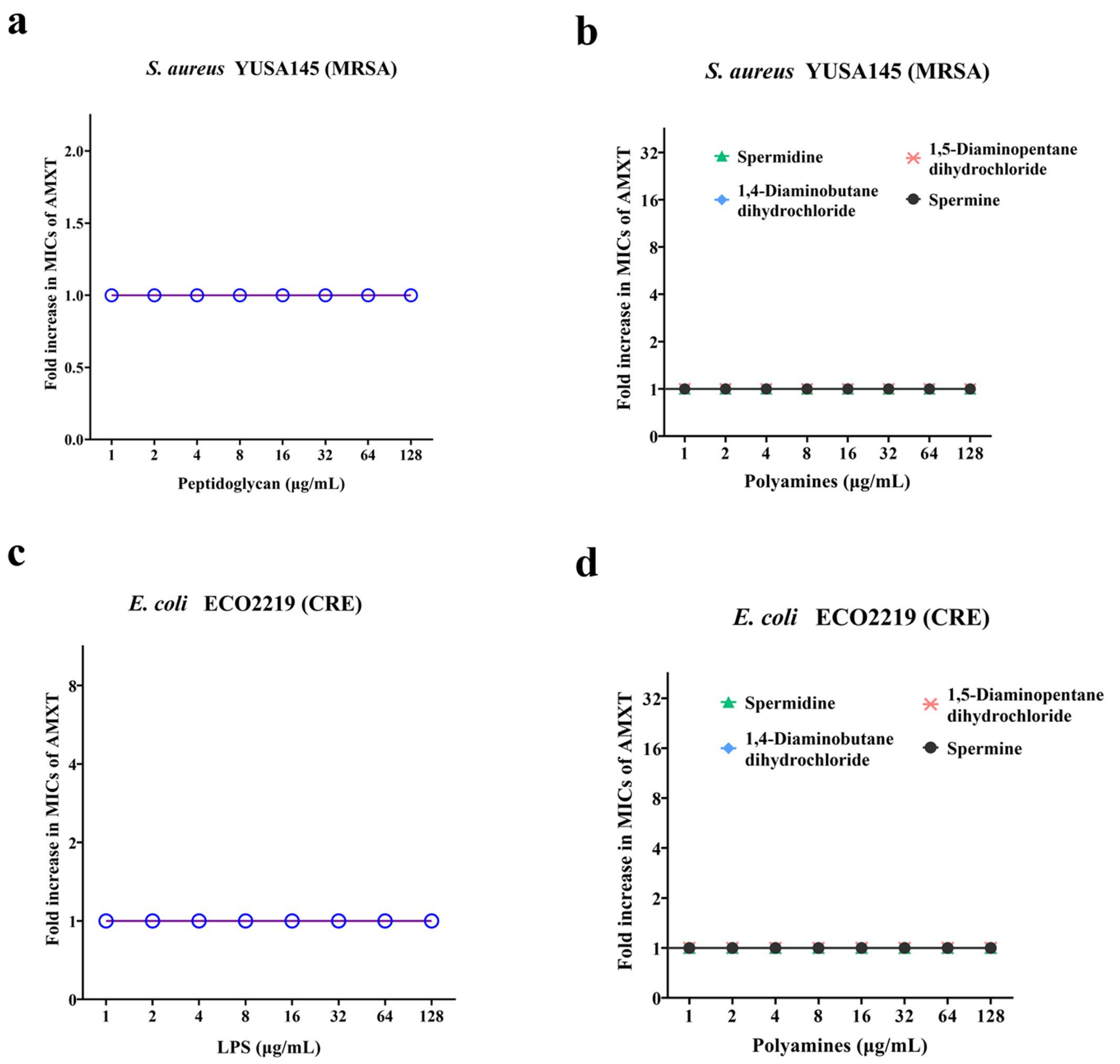

Figure S10. The role of PG, LPS, and polyamines in the antibacterial activity of AMXT-1501. AMXT-1501 MICs against *S. aureus*YUSA145 in the presence of PG (**a**) and four polyamines (spermine, spermidine, 1,4-diaminobutane dihydrochloride and 1,5-diaminopentane dihydrochloride)(**b**). AMXT-1501 MICs against *E. coli* ECO2219 in the presence of LPS (**c**) and the same four polyamines (**d**). Data were obtained from checkerboard microdilution assays (range, 1–128 µM).

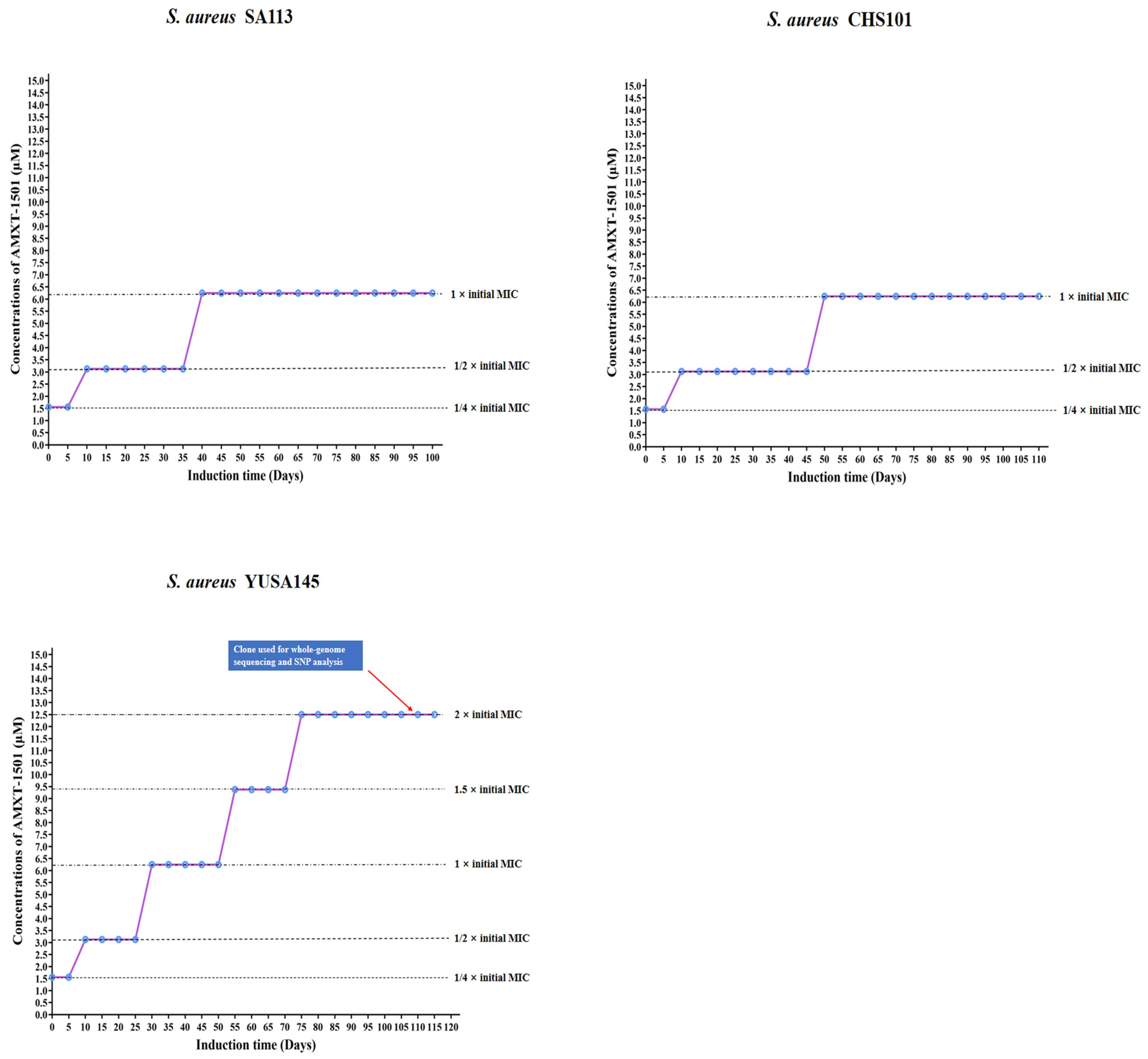

Figure S11. *In vitro* induction of AMXT-1501 non-sensitive clones of *S. aureus*. Three *S. aureus* strains were subcultured serially in TSB containing AMXT-1501. The concentration of AMXT-1501 was increased successively from an initial concentration of 1/4× MIC. Cells were cultured at each concentration for 3–5 passages before being exposed to the next concentration. Isolates from the last passage of each concentration were picked. Clones were selected and identified by matrix-assisted laser desorption ionization time-of-flight mass spectrometry. The MICs of AMXT-1501 were determined again.

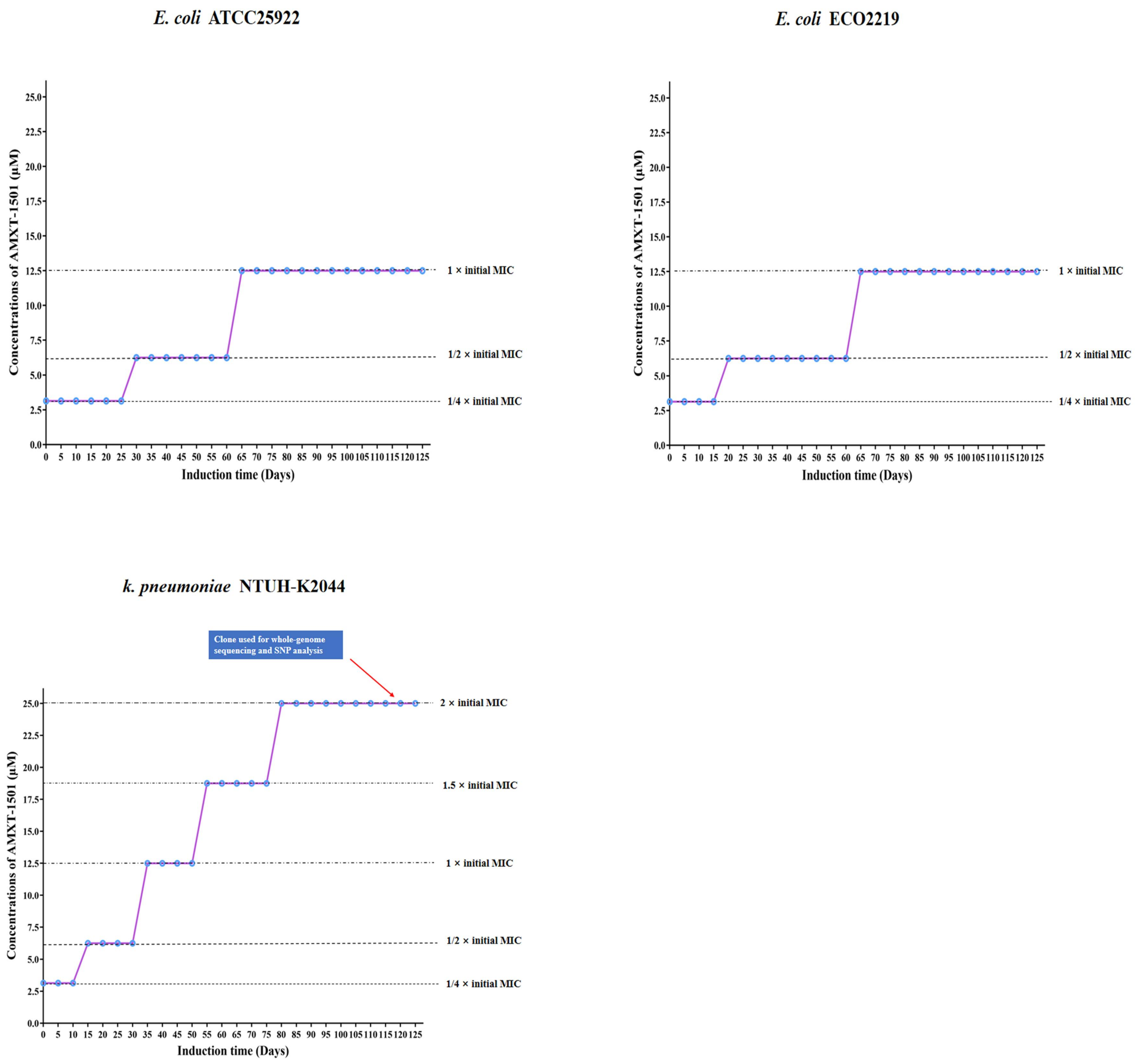

Figure S12. ***In vitro*** **induction of AMXT-1501 non-sensitive clones of *E. coli* and *K. pneumoniae*.** Two *E. coli* and one *K. pneumoniae* strains were subcultured serially in TSB containing AMXT-1501. The concentration of AMXT-1501 was increased successively from an initial concentration of 1/4× MIC. Cells were cultured at each concentration for 3–5 passages before being exposed to the next concentration. Isolates from the last passage of each concentration were picked. Clones were selected and identified by matrix-assisted laser desorption ionization time-of-flight mass spectrometry. The MICs of AMXT-1501 were determined again.

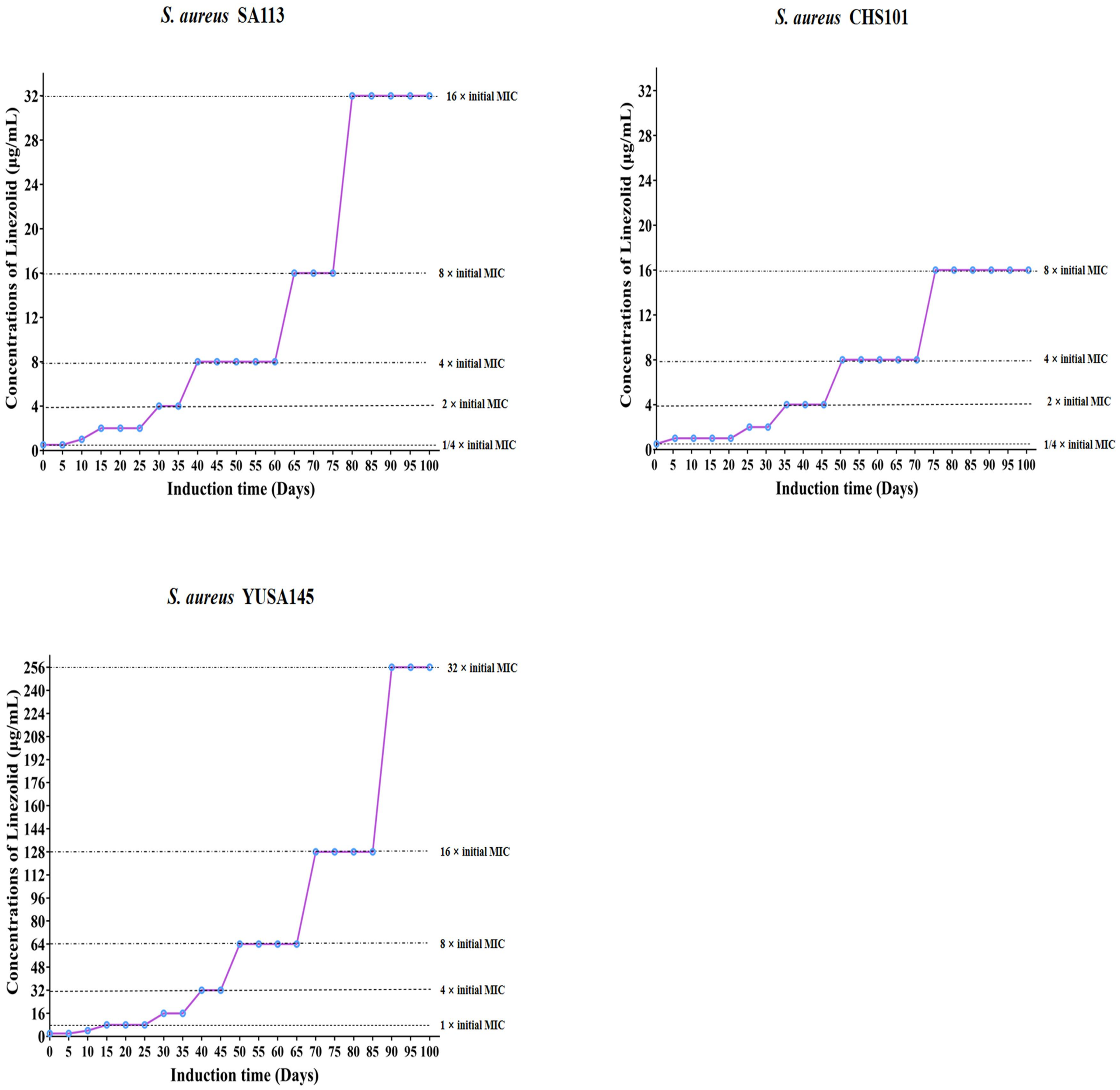

Figure S13. *In vitro* induction of linezolid non-sensitive clones of *S. aureus.* Three *S. aureus* strains were subcultured serially in TSB containing linezolid. The concentration of linezolid was increased successively from an initial concentration of 1/4× MIC. Cells were cultured at each concentration for 3–5 passages before being exposed to the next concentration. Isolates from the last passage of each concentration were picked. Clones were selected and identified by matrix-assisted laser desorption ionization time-of-flight mass spectrometry. The MICs of linezolid were determined again.

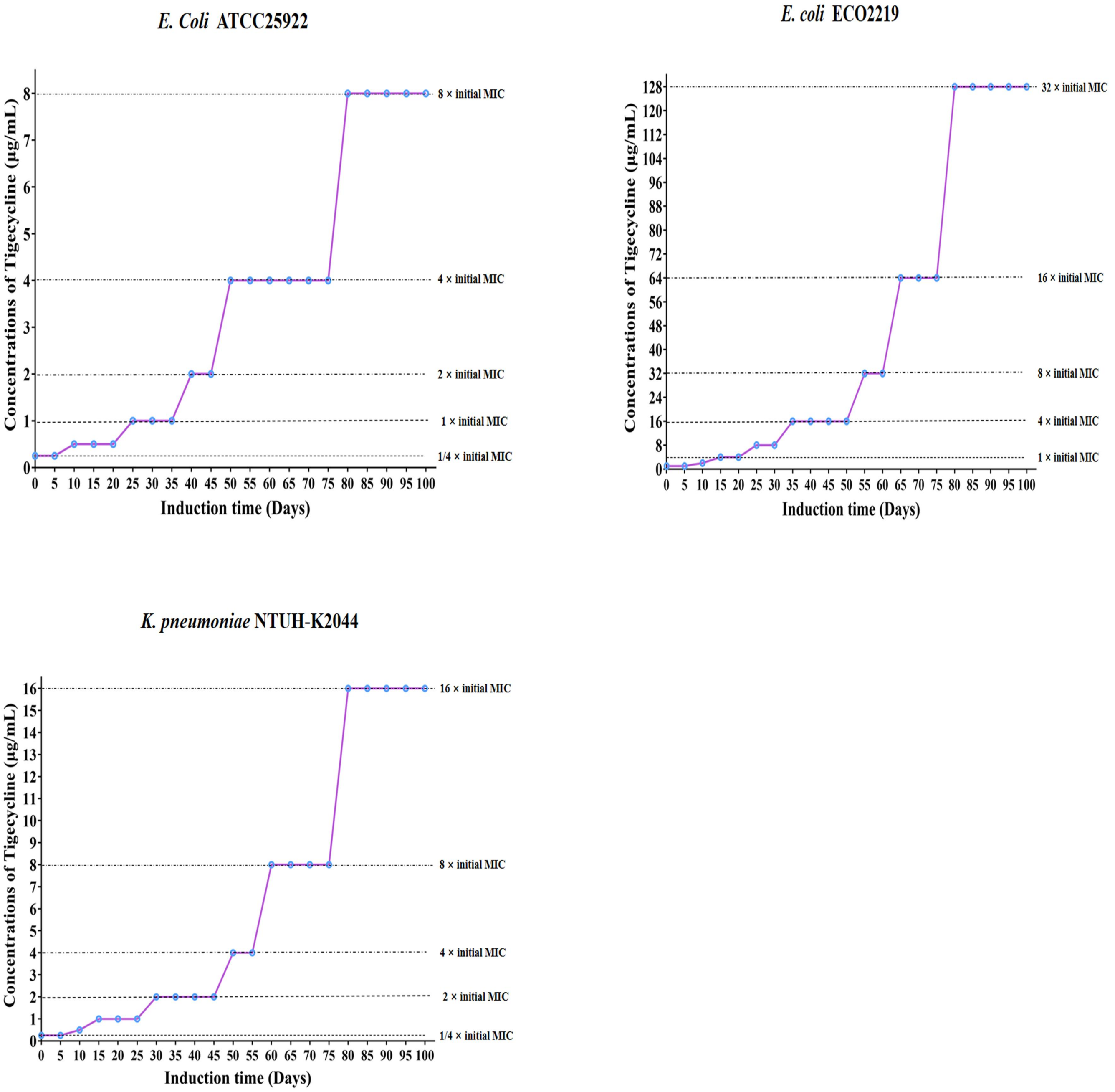

Figure S14. ***In vitro*** **induction of tigecycline non-sensitive clones of** ***E. coli*** **and** ***K. pneumoniae.*** Two *E. coli* and one *K. pneumoniae* strains were subcultured serially in TSB containing tigecycline. The concentration of tigecycline was increased successively from an initial concentration of 1/4× MIC. Cells were cultured at each concentration for 3–5 passages before being exposed to the next concentration. Isolates from the last passage of each concentration were picked. Clones were selected and identified by matrix-assisted laser desorption ionization time-of-flight mass spectrometry. The MICs of tigecycline were determined again.
